# Impact of Antiretroviral Therapy Duration on HIV-1 Infection of T Cells within Anatomic Sites

**DOI:** 10.1128/JVI.01270-19

**Published:** 2020-01-17

**Authors:** Eunok Lee, Susanne von Stockenstrom, Vincent Morcilla, Lina Odevall, Bonnie Hiener, Wei Shao, Wendy Hartogensis, Peter Bacchetti, Jeffrey Milush, Teri Liegler, Elizabeth Sinclair, Hiroyu Hatano, Rebecca Hoh, Ma Somsouk, Peter Hunt, Eli Boritz, Daniel Douek, Remi Fromentin, Nicolas Chomont, Steven G. Deeks, Frederick M. Hecht, Sarah Palmer

**Affiliations:** a The Westmead Institute for Medical Research, University of Sydney, Westmead, New South Wales, Australia; b Department of Microbiology, Tumor and Cell Biology, Karolinska Institutet, Karolinska University Hospital, Stockholm, Sweden; c Advanced Biomedical Computing Center, Leidos Biomedical Research Inc., Frederick National Laboratory for Cancer Research, Frederick, Maryland, USA; d Department of Medicine, University of California San Francisco, San Francisco, California, USA; e Department of Epidemiology and Biostatistics, University of California San Francisco, San Francisco, California, USA; f Human Immunology Section, Vaccine Research Center, National Institute of Allergy and Infectious Diseases, National Institutes of Health, Bethesda, Maryland, USA; g Centre de Recherche du CHUM and Department of Microbiology, Infectiology and Immunology, Université de Montréal, Montreal, Canada; Emory University

**Keywords:** acute/early infection, anatomic sites, CD4^+^ T cell subsets, cellular proliferation, chronic infection, HIV-1, HIV-1 persistence, long-term antiretroviral therapy, single-genome sequencing, single-proviral sequencing

## Abstract

Understanding the impact of antiretroviral therapy (ART) duration on HIV-infected cells is critical for developing successful curative strategies. To address this issue, we conducted a cross-sectional/inter-participant genetic characterization of HIV-1 RNA from pre- and on-therapy plasmas and HIV-1 DNA from CD4^+^ T cell subsets derived from peripheral blood (PB), lymph node (LN), and gut tissues of 26 participants after 3 to 17.8 years of ART. Our studies revealed in four acute/early participants who had paired PB and LN samples a substantial reduction in the proportion of HIV-infected cells per year on therapy within the LN. Extrapolation to all 12 acute/early participants estimated a much smaller reduction in the proportion of HIV-1-infected cells within LNs per year on therapy that was similar to that in the participants treated during chronic infection. LN-derived effector memory T (T_EM_) cells contained HIV-1 DNA that was genetically identical to viral sequences derived from pre- and on-therapy plasma samples. The proportion of identical HIV-1 DNA sequences increased within PB-derived T_EM_ cells. However, the infection frequency of T_EM_ cells in PB was stable, indicating that cellular proliferation that compensates for T cell loss over time contributes to HIV-1 persistence. This study suggests that ART reduces HIV-infected T cells and that clonal expansion of HIV-infected cells maintains viral persistence. Importantly, LN-derived T_EM_ cells are a probable source of HIV-1 genomes capable of producing infectious HIV-1 and should be targeted by future curative strategies.

**IMPORTANCE** HIV-1 persists as an integrated genome in CD4^+^ memory T cells during effective therapy, and cessation of current treatments results in resumption of viral replication. To date, the impact of antiretroviral therapy duration on HIV-infected CD4^+^ T cells and the mechanisms of viral persistence in different anatomic sites is not clearly elucidated. In the current study, we found that treatment duration was associated with a reduction in HIV-infected T cells. Our genetic analyses revealed that CD4^+^ effector memory T (T_EM_) cells derived from the lymph node appeared to contain provirus that was genetically identical to plasma-derived virions. Moreover, we found that cellular proliferation counterbalanced the decay of HIV-infected cells throughout therapy. The contribution of cellular proliferation to viral persistence is particularly significant in T_EM_ cells. Our study emphasizes the importance of HIV-1 intervention and provides new insights into the location of memory T cells infected with HIV-1 DNA, which is capable of contributing to viremia.

## Abstract (precis)

HIV-1 persists as an integrated genome in CD4^+^ memory T cells during effective therapy, and cessation of current treatments results in resumption of viral replication. To date, the impact of antiretroviral therapy duration on HIV-infected CD4^+^ T cells and the mechanisms of viral persistence in different anatomic sites is not clearly elucidated. In the current study, we found that treatment duration was associated with a reduction in HIV-infected T cells. Our genetic analyses revealed that CD4^+^ effector memory T (T_EM_) cells derived from the lymph node appeared to contain provirus that was genetically identical to plasma-derived virions. Moreover, we found that cellular proliferation counterbalanced the decay of HIV-infected cells throughout therapy. The contribution of cellular proliferation to viral persistence is particularly significant in T_EM_ cells. Our study emphasizes the importance of HIV-1 intervention and provides new insights into the location of memory T cells infected with HIV-1 DNA, which is capable of contributing to viremia.

## INTRODUCTION

Antiretroviral therapy (ART) effectively suppresses but does not eradicate HIV-1 infection. HIV-1 persists in a quiescent state within cellular and tissue reservoirs of HIV-1-infected individuals on effective ART (1 – 7). Although ART can effectively halt HIV-1 replication, the virus persists in plasma and cellular reservoirs, and treatment cessation invariably results in the resumption of viral replication (8 – 11). Studies have shown that HIV-1 infection of resting CD4^+^ T cells happens when an activated CD4^+^ T cell becomes infected by HIV-1 but transitions to a quiescent memory state before the infection eliminates the cell, and it has been proposed that HIV-1 has the potential to directly infect resting CD4^+^ T cells (4, 7, 12 – 16).

The number of HIV-1-infected cells is remarkably stable during ART (7, 17). The mechanisms driving the stability of HIV-1 in cellular and tissue reservoirs during suppressive ART are under investigation. The replenishment of these infected cells by low-level ongoing viral replication in anatomic sites containing suboptimal levels of antiviral drugs has been proposed (18 – 22). However, studies of participants on long-term ART have failed to detect HIV-1 evolution, indicating that viral replication during effective ART is limited (23, 24). The presence of identical HIV-1 DNA sequences among diverse viral genetic populations indicates that these persistent HIV-1-infected cells are partially maintained by cellular proliferation (17, 23, 25 – 28). Such cellular mechanisms may be major contributors to the persistence of HIV-1-infected cells and the preservation of proviral HIV-1 DNA during suppressive ART.

The majority of published studies have focused on CD4^+^ T cell subsets within the peripheral blood (PB) when characterizing persistent HIV-1 during ART (29). However, the lymph node (LN) and gut are preferential sites for HIV-1 persistence during therapy (30 – 33). Moreover, it has been found that HIV-1 can utilize a broad range of coreceptors, including CCR6 and CXCR5, indicating that the virus can establish infection in T cells expressing these markers (34, 35). Therefore, our study aims to define the impact of ART duration on the dynamics and genetic composition of the *gag-pol* region (p6 through nucleotides 1 to 900 of the gene encoding reverse transcriptase [p6-RT]) of HIV-1 within a broad range of T cell subsets derived from different anatomic sites. We performed cross-sectional/interparticipant analysis of HIV-1 DNA sequences in CD4^+^ T cell subsets derived from the peripheral blood, lymph node, and gut tissues of 26 participants who had received 3 to 17.8 years of suppressive ART. We modeled the impact of therapy duration on the proportion of HIV-1-infected cells and the genetic nature of the virus to understand the cellular mechanisms contributing to viral persistence during therapy. Moreover, we genetically compared HIV-1 RNA sequences derived from pretherapy and early on-therapy plasma and viral DNA sequences derived from CD4^+^ T cell subsets sorted from the anatomic sites to identify intracellular HIV-1 sources contributing to viremia during ART.

Our study suggested a decline in the proportion of T cells that were HIV-1 infected. We found no substantial accumulation of genetically defective HIV-1 sequences in participants who initiated ART during acute/early and chronic infection, which indicates that the pool of defective viral genomes is established in cells during multiple rounds of HIV-1 replication before viral suppression. Moreover, the genetic comparison of viral populations between plasma and a broad spectrum of CD4^+^ T cell subsets indicated that lymph node-derived CD4^+^ effector memory T (T_EM_) cells are a likely source of HIV-1 genomes capable of producing infectious virus. Furthermore, our in-depth genetic analysis revealed that cellular proliferation contributes to HIV-1 persistence by restoring the overall stability of HIV-1-infected cells despite T cell loss during therapy.

## RESULTS

### HIV-1 infection frequencies of T cells located in different anatomic sites during effective ART.

**FIG 1:**
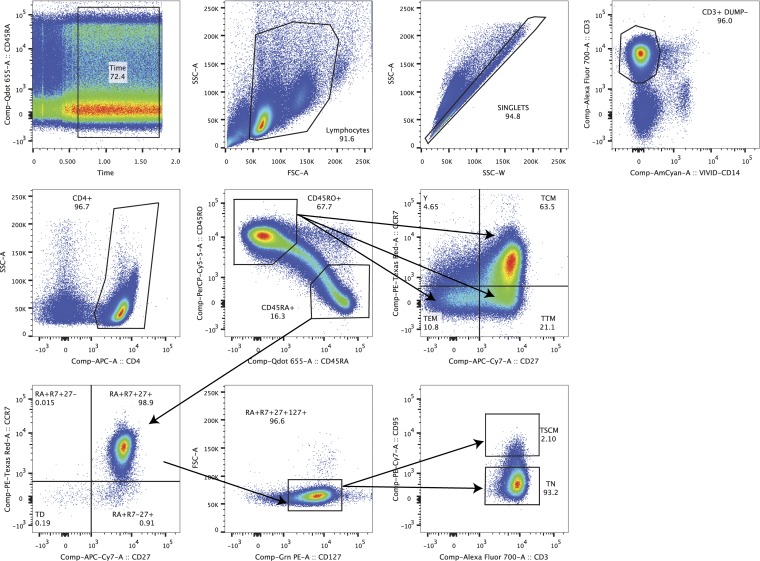
Example of the gating strategy for classical CD4^+^ T cell populations from peripheral blood. Shown is the gating strategy for the CD4^+^ T_N_, T_SCM_, T_CM_, T_TM_, and T_EM_ cell subsets.

**FIG 2:**
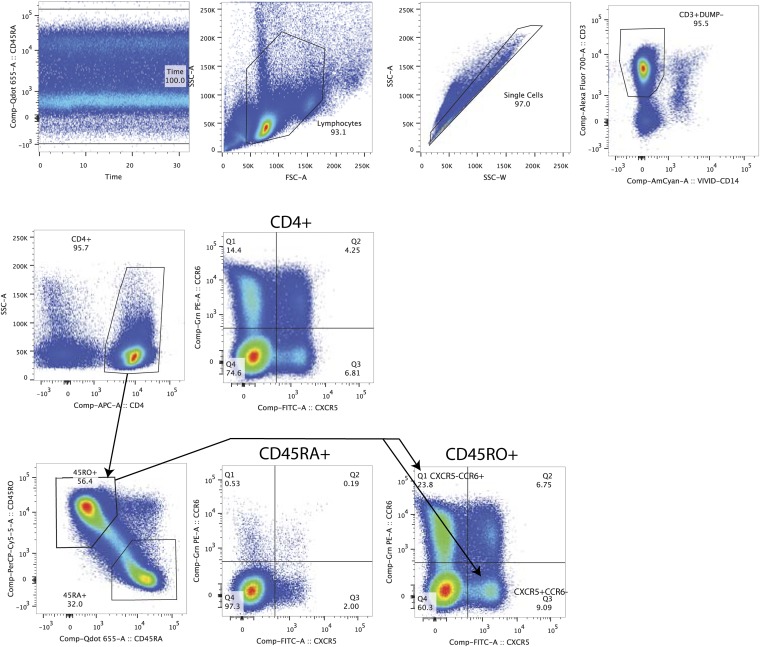
Example of the gating strategy for sorting CXCR5^+^ CCR6^−^ and CXCR5^−^ CCR6^+^ CD4^+^ T cell memory populations from peripheral blood. Shown is the gating strategy for memory lymph node-homing (X5^+^ R6^−^) and memory gut-homing (X5^−^ R6^+^) CD4^+^ T cell subsets.

**FIG 3:**
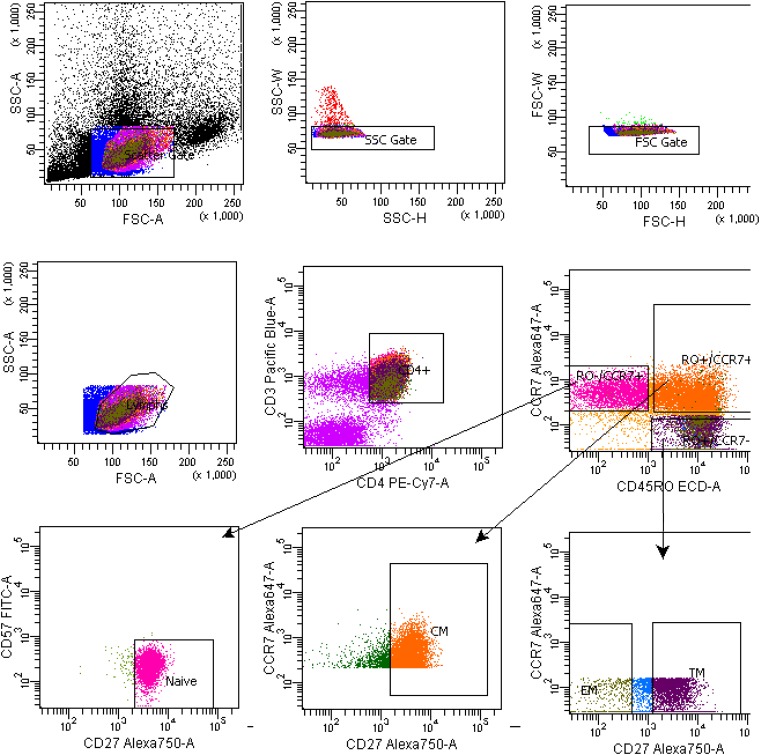
Example of the gating strategy for sorting classical CD4^+^ T cell populations from lymph nodes. Shown is the gating strategy for CD4^+^ T_N_, T_CM_, T_TM_, and T_EM_ cell subsets.

The impact of ART duration on the proportion of HIV-1-infected T cells is not clearly defined. To evaluate the effect of ART duration on the proportion of infected T cells, we performed a cross-sectional/interparticipant analysis of the proportion of HIV-1-infected cells in CD4^+^ T cell subsets sorted from PB, LN, and gut tissues. We sorted a broad range of CD4^+^ T cell subsets from the anatomic sites using their specific cellular markers in 26 participants after they had been on effective ART for 3.0 to 17.8 years: 12 who initiated therapy during acute/early HIV-1 infection (≤6 months of infection before initiation of therapy) (AHI group) and 14 who initiated therapy during chronic HIV-1 infection (≥1 year of infection before initiation of therapy) (CHI group) (Tables 1 to 3 and Fig. 1 to 3). The anatomic regions and cellular subsets were collected after the stated duration of ART for each participant (Table 1). These participants were continuously suppressed during the study, except for one participant who had a viral rebound at the time of sampling (Table 1). We used a previously described maximum-likelihood method to estimate the proportion of cells infected within each T cell subset and within each anatomic site (23). The influence of ART duration on the proportions of infected cells are presented as a fold effect per year of ART. We estimated the fold differences in the proportions of cells infected between earlier and later time points. A fold effect per year of ART greater than 1 indicates that a higher proportion of HIV-1-infected T cells is associated with each additional year on therapy, while a value of 1 means a stable proportion of infected T cells during treatment. A fold effect per year of less than 1 indicates that a lower proportion of infected T cells is associated with each additional year of ART. The statistical significance indicates the evidence for increase (a fold effect per year of greater than 1) or decrease (a fold effect per year of less than 1) versus the null hypothesis of no change over the duration of ART (a fold effect per year of 1). Furthermore, we estimated the impact of ART duration on the proportions of infected cells when at least four participants contributed to the fold effect per year on ART within each T cell subset and tissue.

**TABLE 1:**
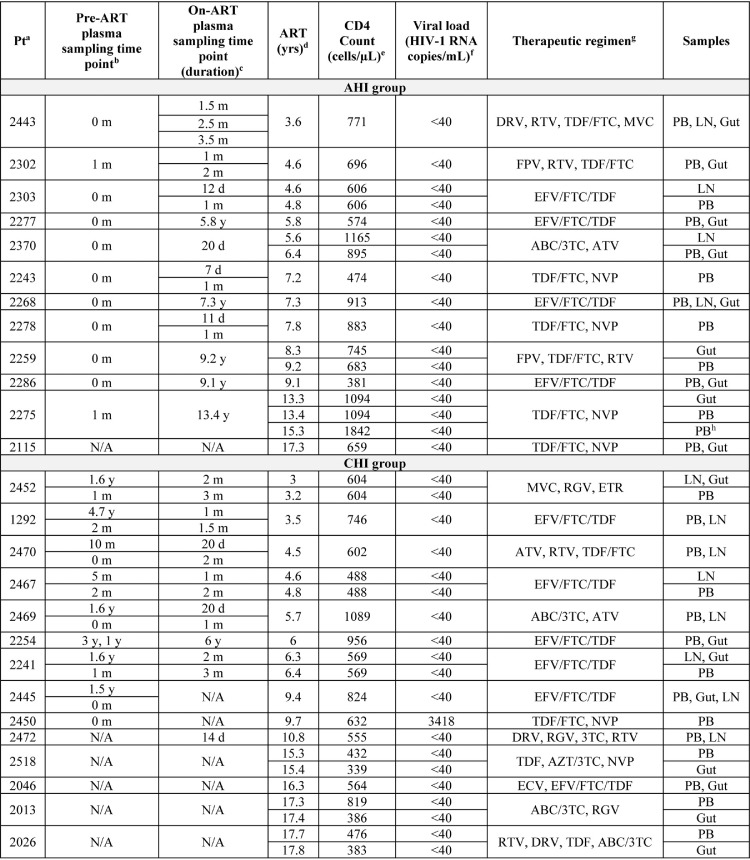
Participant demographics and clinical samples

a Pt, participant.

b Years (y) and months (m) before ART initiation. Only the CHI group was used for analysis. NA, not applicable.

c Years, months, or days (d) after ART initiation. Only the CHI group was used for analysis. NA, not applicable.

d Duration on ART at the time of sample isolation.

e CD4 cell count at the time of sample isolation.

f Viral load at the time of sample isolation.

g Therapeutic regimen at the time of sample isolation. 3TC, lamivudine; ABC, abacavir; ATV, atazanavir; AZT, zidovudine; DRV, darunavir; ECV, entecavir; EFV, efavirenz; ETR, etravirine; FPB, fosamprenavir; FTC, emtricitabine; MVC, maraviroc; NVP, nevirapine; RGV, raltegravir; RTV, ritonavir; TDF, tenofovir.

h Excluded from analyses of the fold effect of the proportion of cells infected for cross-sectional analysis.

**TABLE 2:** Numbers of CD4^+^ T cells used for analysis

Pt^*a*^	PB							LN				Gut			
	T_N_	T_SCM_	T_CM_	T_EM_	T_EM_	R6^+^^*a*^	X5^+^^*a*^	T_N_	T_CM_	T_TM_	T_EM_	T_N_	T_CTM_	T_EM_	CD4
AHI															
2243	3,014,850	NA	3,809,056	4,673,276	2,404,690	NA	NA	NA	NA	NA	NA	NA	NA	NA	NA
2302	1,330,864	NA	797,531	296,884	458,564	NA	NA	NA	NA	NA	NA	17,358	182,989	97,127	NA
2303	330,864	NA	375,309	335,246	261,200	NA	NA	2,022,983	59,288	59,318	592,693	NA	NA	NA	NA
2277	1,200,000	NA	100,960	45,432	55,479	NA	NA	NA	NA	NA	NA	237	21,115	10,603	NA
2370	3,000,000	NA	3,600,000	6,000,000	944,444	NA	NA	1,674,001	1,040,000	2,196,000	388,000	3,816	81,863	76,234	NA
2443	975,309	NA	906,173	822,222	866,667	NA	NA	154,854	84,387	97,637	36,802	8,578	153,252	106,970	NA
2268	6,500,000	NA	6,000,000	5,400,000	1,500,000	NA	NA	676,184	687,452	1,388,581	282,959	2,094	40,141	30,937	NA
2278	1,476,049	NA	2,000,259	1,512,837	800,539	NA	NA	NA	NA	NA	NA	NA	NA	NA	NA
2259	6,000,000	NA	2,330,864	59,483	332,905	NA	NA	NA	NA	NA	NA	18,670	44,651	35,267	NA
2286	5,212,158	NA	256,498	424,160	26,475	NA	NA	NA	NA	NA	NA	76	8,036	9,645	NA
2275	10,429,091	8,364	5,851,628	1,210,685	2,255,922	249,158	302,761	NA	NA	NA	NA	3,709	79,197	50,592	NA
2115	4,404,848	8,444	1,694,276	439,394	1,515,152	311,448	267,116	NA	NA	NA	NA	NA	NA	NA	18,182
CHI															
2452	108,642	NA	88,889	102,058	108,724	NA	NA	171,016	143,775	119,718	180,179	2,867	65,317	40,361	NA
1292	3,008,265	NA	92,347	118,552	454,644	NA	NA	4,192,593	18,999	32,118	26,919	NA	NA	NA	NA
2470	497,456	NA	227,579	503,719	227,322	NA	NA	47,438	70,074	50,643	18,696	NA	NA	NA	NA
2467	1,399,891	NA	797,687	478,109	399,501	NA	NA	402,207	72,717	96,955	13,476	NA	NA	NA	NA
2469	2,002,843	NA	237,283	139,048	356,717	NA	NA	316,545	48,391	66,250	10,844	NA	NA	NA	NA
2254	1,160,165	NA	42,798	52,675	70,584	NA	NA	NA	NA	NA	NA	62	11,274	11,499	NA
2241	59,259	NA	118,519	87,259	122,902	NA	NA	115,681	69,333	147,793	20,622	2,700	24,200	43,700	NA
2445	2,533,333	NA	111,111	83,951	50,405	NA	NA	2,463,674	479,499	353,437	98,905	544	10,998	51,545	NA
2450	1,133,333	NA	250,206	22,359	59,991	NA	NA	NA	NA	NA	NA	NA	NA	NA	NA
2472	6,185,766	NA	731,875	771,811	1,393,073	NA	NA	361,722	264,048	656,120	136,488	NA	NA	NA	NA
2518	1,143,939	8,613	1,869,809	2,047,138	6,810,236	278,788	454,545	NA	NA	NA	NA	NA	NA	NA	18,182
2046	196,566	4,175	54,471	9,577	38,908	95,623	99,663	NA	NA	NA	NA	NA	NA	NA	18,182
2013	699,717	69,662	787,027	539,237	1,836,913	266,264	132,300	NA	NA	NA	NA	NA	NA	NA	153,535
2026	384,000	218,182	1,097,222	171,212	970,202	137,535	185,152	NA	NA	NA	NA	NA	NA	NA	90,909
Avg	2,384,711	52,907	1,267,755	975,790	900,821	223,136	240,256	1,049,908	253,164	438,714	150,549	5,059	60,253	47,040	59,798

a Pt, participant; R6^+^, X5^−^ R6^+^ T cell subset; X5^+^, X5^+^ R6^−^ T cell subset.

**TABLE 3:** Cellular markers used for a broad spectrum of CD4^+^ T cell subsets sorted from PB, LNs, and gut

Anatomic site	CD4^+^ T cell subset	Cell markers	Pt IDs^*a*^
PB	T_N_	CD3^+^ CD4^+^ CD45RO^−^ CCR7^+^ CD27^+^ CD57^−^	2243, 2302, 2303, 2277, 2370, 2443, 2268, 2278, 2259, 2286, 2452, 1292, 2470, 2467, 2469, 2254, 2241, 2445, 2450, 2472
		CD3^+^ CD4^+^ CD45RA^+^ CD45RO^−^ CCR7^+^ CD27^+^ CD127^+^ CD95^−^	2275, 2115, 2518, 2046, 2013, 2026
	T_SCM_	CD3^+^ CD4^+^ CD45RA^+^ CD45RO^−^ CCR7^+^ CD27^+^ CD127^+^ CD95^+^	2275, 2115, 2518, 2046, 2013, 2026
	T_CM_	CD3^+^ CD4^+^ CD45RO^+^ CCR7^+^ CD27^+^	2243, 2302, 2303, 2277, 2370, 2443, 2268, 2278, 2259, 2286, 2452, 1292, 2470, 2467, 2469, 2254, 2241, 2445, 2450, 2472
		CD3^+^ CD4^+^ CD45RA^−^ CD45RO^+^ CCR7^+^ CD27^+^	2275, 2115, 2518, 2046, 2013, 2026
	T_TM_	CD3^+^ CD4^+^ CD45RO^+^ CCR7^−^ CD27^+^	2243, 2302, 2303, 2277, 2370, 2443, 2268, 2278, 2259, 2286, 2452, 1292, 2470, 2467, 2469, 2254, 2241, 2445, 2450, 2472
		CD3^+^ CD4^+^ CD45RA^−^ CD45RO^+^ CCR7^−^ CD27^+^	2275, 2115, 2518, 2046, 2013, 2026
	T_EM_	CD3^+^ CD4^+^ CD45RO^+^ CCR7^−^ CD27^−^	2243, 2302, 2303, 2277, 2370, 2443, 2268, 2278, 2259, 2286, 2452, 1292, 2470, 2467, 2469, 2254, 2241, 2445, 2450, 2472
		CD3^+^ CD4^+^ CD45RA^−^ CD45RO^+^ CCR7^−^ CD27^−^	2275, 2115, 2518, 2046, 2013, 2026
	X5^+^ R6^−^	CD3^+^ CD4^+^ CD45RA^−^ CD45RO^+^ CXCR5^+^ CCR6^−^	2275, 2115, 2518, 2046, 2013, 2026
	X5^−^ R6^+^	CD3^+^ CD4^+^ CD45RA^−^ CD45RO^+^ CXCR5^−^ CCR6^+^	2275, 2115, 2518, 2046, 2013, 2026
LNs	T_N_	CD3^+^ CD4^+^ CD45RO^−^ CCR7^+^ CD27^+^ CD57^−^	2303, 2370, 2443, 2268, 2452, 1292, 2470, 2467, 2469, 2241, 2445, 2472
	T_CM_	CD3^+^ CD4^+^ CD45RO^+^ CCR7^+^ CD27^+^	2303, 2370, 2443, 2268, 2452, 1292, 2470, 2467, 2469, 2241, 2445, 2472
	T_TM_	CD3^+^ CD4^+^ CD45RO^+^ CCR7^−^ CD27^+^	2303, 2370, 2443, 2268, 2452, 1292, 2470, 2467, 2469, 2241, 2445, 2472
	T_EM_	CD3^+^ CD4^+^ CD45RO^+^ CCR7^−^ CD27^−^	2303, 2370, 2443, 2268, 2452, 1292, 2470, 2467, 2469, 2241, 2445, 2472
Gut	T_N_	CD3^+^ CD4^+^ CD45RO^−^ CD27^+^	2302, 2277, 2370, 2443, 2268, 2259, 2286, 2275, 2452, 2254, 2241, 2445
	T_CTM_	CD3^+^ CD4^+^ CD45RO^+^ CD27^+^	2302, 2277, 2370, 2443, 2268, 2259, 2286, 2275, 2452, 2254, 2241, 2445
	T_EM_	CD3^+^ CD4^+^ CD45RO^+^ CD27^−^	2302, 2277, 2370, 2443, 2268, 2259, 2286, 2275, 2452, 2254, 2241, 2445
	CD4^+^	CD3^+^ CD4^+^ CD8^−^ CD14^−^	2115, 2518, 2046, 2013, 2026

a Identifiers (IDs) of participants included in the particular cell sorting.

**FIG 4:**
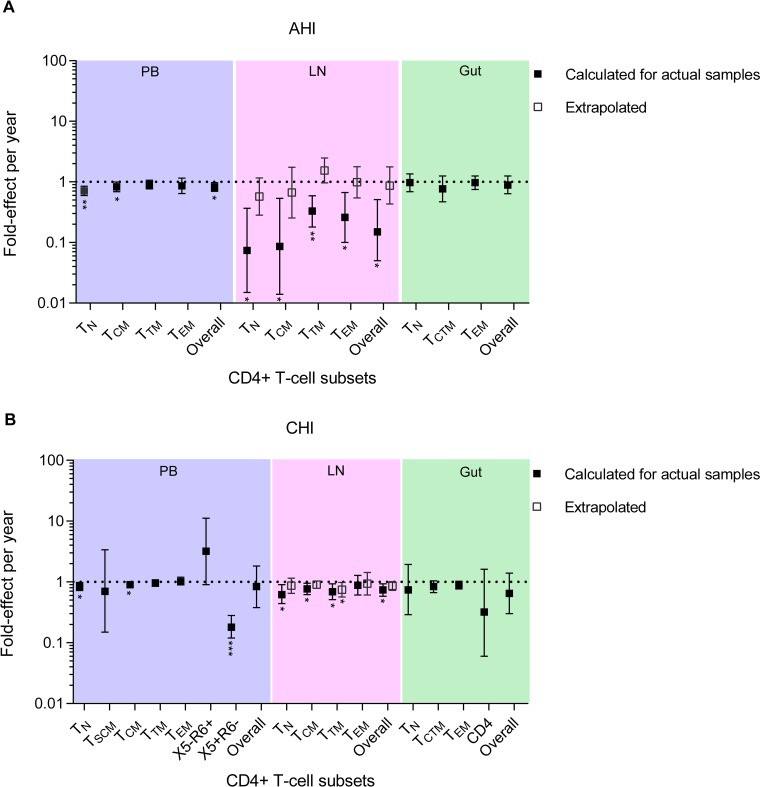
Fold effect of infection frequency during each year on ART. Shown is the fold effect per year of therapy on infection frequency in a broad range of CD4^+^ T cell subsets sorted from PB (blue shading), LN (pink shading), and gut (green shading) tissues obtained from the AHI group (A) and the CHI group (B). The fold effect per year on therapy is a multiplicative effect that is equivalent to a fold change in the proportion of HIV-1-infected T cells from earlier to later time points during ART. For the LN, the fold effect per year on ART within 4 actual participant samples (with data from both PB and LNs) in the AHI group is indicated by the solid squares. The extrapolated fold effect per year on ART represents all 12 participants in the AHI group (open squares). For the LNs of the CHI group, the fold effect per year on ART derived from 8 actual participant samples (with data from both PB and LNs) is indicated by the solid squares, and the extrapolated fold effect per year of therapy for all 14 participants is indicated by the open squares. For each T cell subset, the fold effect per year on therapy was estimated when samples were available from at least 4 participants. The PB sample obtained from participant 2275 after 15.3 years of therapy was excluded from this cross-sectional analysis (Table 1). The error bars indicate the 95% confidence intervals of the infection frequency fold effect per year. *, *P* < 0.05; **, *P* < 0.01; ***, *P* < 0.001. The effects were estimated by negative binomial regression.

In PB-derived T cells from the AHI group, each additional year of ART was associated with an overall lower proportion of HIV-1-infected T cells that was statistically significant (fold effect = 0.82/year; 95% confidence interval [CI] = 0.70 to 0.97; *P* = 0.034) (Fig. 4A). This equates to an 18% reduction in the proportion of cells infected during each additional year on therapy. The fold effect on the proportion of cells infected per year in naive (T_N_), central memory (T_CM_), transitional memory (T_TM_), and effector memory (T_EM_) T cell subsets sorted from the AHI group ranged from 0.71 to 0.90, all indicating that a lower proportion of infected T cells was associated with each additional year of therapy. This lower proportion of HIV-1-infected T cells was statistically significant in T_N_ and T_CM_ cells. As we obtained stem cell memory T (T_SCM_) cells from only two participants from the AHI group, we were not able to examine the association between the ART duration and the proportion of infected T cells within this T cell subset (Table 2). For the CHI group, the fold effect per year of ART within the infected T cells from the PB was similar to that for the AHI group (fold effect = 0.84/year; 95% CI = 0.38 to 1.82; *P* = 0.59) but not statistically significant (Fig. 4B). The fold effects on the proportion of infected T cells associated with each additional year on therapy in T_N_, T_CM_, T_TM_, and T_EM_ cells derived from the CHI group were also similar to those from the AHI group. The PB-derived T_SCM_ cells were obtained from four participants in the CHI group. The reduction in the proportions of infected T_SCM_ cells during each additional year on therapy was not statistically significant (fold effect = 0.70/year; 95% CI = 0.15 to 3.37; *P* = 0.56). Of interest, T_EM_ cells derived from the CHI group had strong evidence for a stable proportion of HIV-1-infected T cells during each additional year on therapy, with a fold effect of 1.01 per year (95% CI = 0.87 to 1.17). However, CXCR5^+^ CCR6^−^ (X5^+^ R6^−^) memory CD4^+^ T cells derived from the CHI group had a substantially lower proportion of infected T cells associated with each additional year of ART.

The LN data were derived from 4 participants from the AHI group, with samples obtained from 3.6 to 7.3 years of therapy (Tables 1 and 2). For 8 participants from the CHI group, the LN samples were obtained from 3 to 10.8 years of therapy (Tables 1 and 2). For these LN samples, we found an overall lower proportion of HIV-1-infected T cells associated with each additional year on therapy, with a fold effect of 0.15/year and 0.74/year for the AHI and CHI groups, respectively (Fig. 4A and B). For the AHI group, the fold effect per year on therapy was influenced by all the T cell subsets sorted from LNs (Fig. 4A). For the CHI group, all the T cell subsets sorted from LNs also had a lower proportion of infected cells per year on ART, with a fold effect of 0.62 to 0.88 per year on therapy (Fig. 4B). Overall, our results provide some evidence that HIV-1-infected T cells decay substantially during each additional year of therapy within the LN-derived T cell subsets we sorted from 4 participants in the AHI group compared to the 8 participants from the CHI group.

We also found a strong correlation between the proportion of HIV-1-infected T cells sorted from LNs and PB within the acute/early participants for whom we had both LN and PB samples available (LN and PB correlations within T cell subsets were 0.98 to 0.99 [Table 4]). Among the chronic participants who had both samples available, we also found a strong correlation between LNs and PB within the T_N_ and T_CM_ cell subsets (0.93 and 0.96, respectively). We used mixed-effects models of both PB and LN data together to obtain extrapolated fold effects per year in LN-derived T cell subsets. These models exploit the PB-LN correlations to estimate what the effects would have been for all 12 AHI and all 14 CHI participants. These models estimated an overall 13% reduction in the proportion of infected T cells during each additional year on therapy in LNs of the AHI group, with a fold effect of 0.87/year (95% CI = 0.43 to 1.77) (Fig. 4A). This lower proportion of cells infected per year on ART was influenced by T_N_ (fold effect = 0.57/year) and T_CM_ (fold effect = 0.67/year) cells. In the CHI group, the fold effects per year on therapy derived from the extrapolation of T cell subsets located in the LNs were comparable to those measured within the actual samples (0.86/year and 0.74/year, respectively) (Fig. 4B). This equates to a 14% reduction in the proportion of infected cells in the CHI group and was comparable to the overall reduction in LNs derived from the AHI group.

**TABLE 4:** Correlations between log proportions of HIV-1-infected T cells within PB, LNs, and gut

Correlation	Estimate	95% CI		*P* value
		Lower	Upper	
AHI group				
PB T_N_ with LN T_N_	0.98	0.41	1.00	0.16
PB T_CM_ with LN T_CM_	0.99	0.54	1.00	0.012
PB T_TM_ with LN T_TM_	0.99	0.71	1.00	0.0068
PB T_EM_ with LN T_EM_	0.99	0.50	1.00	0.012
PB T_N_ with gut T_N_	−0.31	−0.86	0.58	0.95
PB T_CM_ and T_TM_ with gut T_CTM_	0.75	0.10	0.95	0.032
PB T_EM_ with gut T_EM_	0.62	−0.08	0.91	0.076
CHI group				
PB T_N_ with LN T_N_	0.93	0.64	0.99	0.0009
PB T_CM_ with LN T_CM_	0.96	0.78	0.99	0.0002
PB T_TM_ with LN T_TM_	0.68	−0.05	0.94	0.065
PB T_EM_ with LN T_EM_	0.34	−0.48	0.84	0.40
PB T_CM_ and T_TM_ with gut T_CTM_	−0.45	−0.99	0.90	0.55
PB T_EM_ with gut T_EM_	−0.46	−0.99	0.90	0.54

To assess the association between the ART duration and the proportion of infected cells within the gut tissue, we included nine and eight participants from the AHI and CHI groups, respectively (Tables 1 and 2). In the gut, the change in the proportion of HIV-1-infected T cells, as measured by fold effect, across all T cell subsets was similar to those for PB and LNs (AHI overall = 0.89/year; 95% CI = 0.64 to 1.25; *P* = 0.29; CHI overall = 0.65/year; 95% CI = 0.30 to 1.40; *P* = 0.17) but was not statistically significant for both the AHI and CHI groups (Fig. 4A and B). We also performed a correlation analysis within paired T cell subsets between gut and PB (Table 4). This correlation analysis was performed on participants who had more than 1,000 cells within each T cell subset derived from the gut (Table 2). In the AHI group, we found a moderate correlation with PB-derived T cell subsets within central/transitional memory T (T_CTM_) and T_EM_ cells sorted from the gut. For the CHI group, the correlations between gut and PB within T_CTM_ and T_EM_ cells were 0.79 and 0.93, respectively. However, only the T_EM_ cell values were statistically significant.

**FIG 5:**
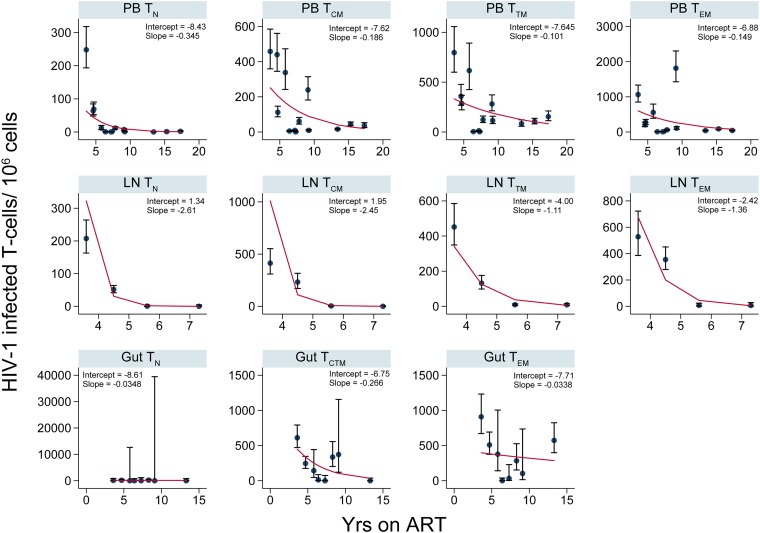
Numbers of HIV-1-infected T cells per million over years on ART (AHI group). The numbers of HIV-1-infected cells per million within CD4^+^ T cell subsets sorted from PB, LNs, and gut are shown. The error bars indicate the 95% confidence interval for each point. The red lines are derived from the fitted values calculated using the following equation at the sampling time points: *y* = 1,000,000 × exp[intercept + slope × (years on ART)]. The coefficients (intercept and slope) used to derive the fitted values for each T cell subset sorted from an anatomic site are shown.

**FIG 6:**
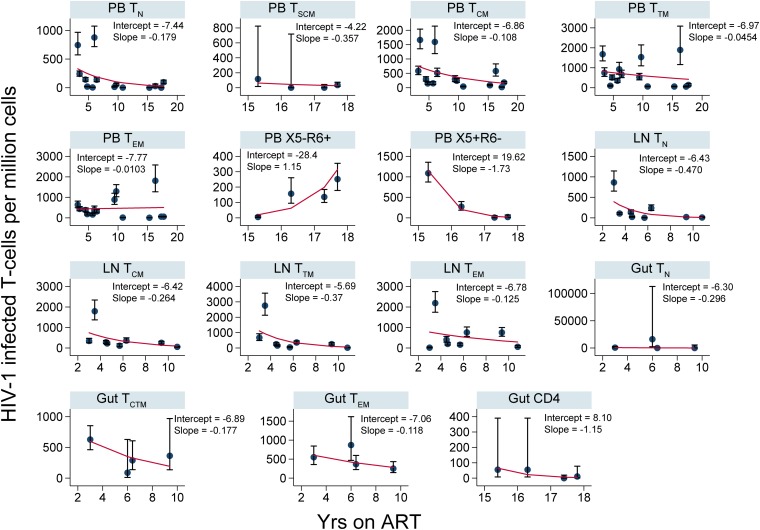
Numbers of HIV-1-infected T cells per million over years on ART (CHI group). The numbers of HIV-1-infected cells per million within CD4^+^ T cell subsets sorted from PB, LNs, and gut are shown. The error bars indicate the 95% confidence interval for each point. The red lines are derived from the fitted values calculated using the following equation at the sampling time points: *y* = 1,000,000 × exp[intercept + slope × (years on ART)]. The coefficients (intercept and slope) used to derive the fitted values for each T cell subset sorted from an anatomic site are shown.

To investigate the changes in cellular infection frequencies within each anatomic site in absolute terms, we determined the number of HIV-1-infected T cells per million within each T cell subset derived from PB, LNs, and gut for all participants (Fig. 5 and 6). These data recapitulated the results of the fold effect analysis per year on ART within each T cell subset.

### Defective HIV-1 DNA in the p6-RT region during therapy.

HIV-1 replication is characterized by rapid and highly error-prone reverse transcription, which lacks proofreading capacity and causes genetic defects in viral genomes (15, 36 – 40). Also, an antiviral mechanism called G-to-A hypermutation renders in-frame stop codons and produces replication-deficient HIV-1 DNA during reverse transcription (15, 41, 42). These genetic defects have been identified in memory T cells during therapy (43 – 45). However, the impact of ART duration on the accumulation of genetically defective HIV-1 DNA is unclear. Therefore, we used a mixed-effects logistic regression model to calculate the proportion of genetically defective HIV-1 DNA p6-RT sequences during each additional year of therapy. Included in this analysis were 3,963 intracellular HIV-1 p6-RT sequences isolated from PB, LNs, and gut (Tables 5 and 6). HIV-1 sequences were defined as genetically defective due to insertions and/or deletions causing a frameshift, G-to-A hypermutations, stop codons, and/or internal deletions.

**TABLE 5:** Numbers of HIV-1 sequences from plasma samples, CD4^+^ T cell subsets, and anatomic sites (AHI group)

Pt^*a*^	Sequence type	Plasma^*a*^		PB^*a*^								LN					Gut				
		PT	OT	Total	T_N_	T_SCM_	T_CM_	T_TM_	T_EM_	R6^+^	X5^+^	Total	T_N_	T_CM_	T_TM_	T_EM_	Total	T_N_	T_CTM_	T_EM_	CD4
2243	Defective	5		19	0		9	9	1												
	Overall	37	0	83	1		30	35	17												
2302	Defective	1	0	30	3		9	5	13								14	0	6	8	
	Overall	35	0	112	31		26	28	27								54	3	24	27	
2303	Defective	3	0	21	2		9	8	2			20	2	4	5	9					
	Overall	29	12	95	27		25	21	22			123	31	30	30	32					
2277^*b*^	Defective	1	1	27	1		12	6	8								3	0	1	2	
	Overall	36	6	90	13		23	27	27								6	0	2	4	
2370	Defective	0	0	7	0		2	5	0			3	0	0	2	1	0	0	0	0	
	Overall	35	0	74	5		26	28	15			26	1	4	19	2	1	0	1	0	
2443	Defective	1	0	23	7		3	4	9			16	1	6	1	8	19	1	8	10	
	Overall	38	1	93	26		18	20	29			85	25	21	13	26	50	1	22	27	
2268^*b*^	Defective	2		3	0		0	2	1			2	0	0	2	0	0	0	0	0	
	Overall	36		25	1		2	10	12			10	0	0	9	1	1	0	0	1	
2278	Defective	5	0	15	3		3	5	4												
	Overall	33	0	90	24		23	24	19												
2259^*b*^	Defective	5	0	32	3		7	14	8								11	0	6	5	
	Overall	47	0	91	4		21	28	38								23	3	11	9	
2286^*b*^	Defective	7	0	16	2		7	5	2								0	0	0	0	
	Overall	48	1	105	23		26	26	30								3	0	2	1	
2275^*b*^	Defective	2	0	6	0	0	2	1	3	0	0						1	0	0	1	
	Overall	30	3	234	5	0	62	63	57	33	14						29	0	0	29	
2115	Defective			55	3	0	7	10	14	8	13						6				6
	Overall			124	8	0	18	23	25	22	28						7				7
Total	Defective	32	1	254	24	0	70	74	65	8	13	41	3	10	10	18	54	1	21	26	6
	Overall	404	23	1216	168	0	300	333	318	55	42	244	57	55	71	61	174	7	62	98	7

a Pt, participant; PT, pretherapy plasma; OT, on-therapy plasma; R6^+^, X5^−^ R6^+^ T cell subset; X5^+^, X5^+^ 6^−^ T cell subset.

b Source of HIV-1 RNA sequences (17, 23).

**TABLE 6:** Numbers of HIV-1 sequences from plasma samples, CD4^+^ T cell subsets, and anatomic sites (CHI group)

Pt^*a*^	Sequence type	Plasma^*a*^		PB^*a*^								LN					Gut				
		PT	OT	Total	T_N_	T_SCM_	T_CM_	T_TM_	T_EM_	R6^+^	X5^+^	Total	T_N_	T_CM_	T_TM_	T_EM_	Total	T_N_	T_CTM_	T_EM_	CD4
2452	Defective	3	0	19	6		4	2	7			11	3	5	3	0	6	1	2	3	
	Identical			45	2		0	22	21			9	0	0	9	0	16	0	8	8	
	Unique			74	28		26	7	13			77	29	29	16	3	34	2	23	9	
	Overall	65	7	125	30		29	30	36			91	29	30	27	5	52	2	33	17	
1292	Defective	3	1	18	3		6	5	4			22	5	8	8	1					
	Identical			14	4		2	0	8			26	6	2	5	13					
	Unique			85	27		26	17	15			91	28	27	22	14					
	Overall	58	30	110	34		31	21	24			125	36	30	29	30					
2470	Defective	9	0	14	4		7	1	2			22	3	8	10	1					
	Identical			25	5		4	3	13			21	4	2	9	6					
	Unique			46	8		15	11	12			47	11	14	15	7					
	Overall	71	3	85	18		21	19	27			86	20	22	26	18					
2467	Defective	1	3	27	6		8	6	7			19	4	9	3	3					
	Identical			19	4		2	0	13			13	4	3	2	4					
	Unique			73	16		24	18	15			41	1	20	12	8					
	Overall	70	36	111	23		29	27	32			67	9	30	14	14					
2469	Defective	4	0	19	3		1	3	12			9	1	0	1	7					
	Identical			30	0		3	8	19			5	0	2	0	3					
	Unique			59	14		21	10	35			19	1	2	3	13					
	Overall	63	7	96	14		28	19	35			29	1	6	3	19					
2254^*b*^	Defective	12	0	16	1		4	8	3								0	0	0	0	
	Identical			14	5		0	0	9								2	0	0	2	
	Unique			70	16		20	20	14								5	1	1	3	
	Overall	91	3	90	22		22	22	24								8	1	1	6	
2241	Defective	3	0	6	0		2	2	2			15	6	4	3	2	0	0	0	0	
	Identical			33	0		3	22	8			21	2	6	4	9	8	0	0	8	
	Unique			79	26		26	11	16			107	28	33	27	19	10	0	5	5	
	Overall	68	1	128	33		31	34	30			134	35	35	31	33	20	0	6	14	
2445^*b*^	Defective	10		32	6		6	6	14			15	3	3	4	5	5	0	1	4	
	Identical			30	7		0	6	17			14	0	0	2	12	0	0	0	2	
	Unique			64	20		22	16	6			82	23	25	23	11	10	0	3	7	
	Overall	77		106	27		26	26	27			108	27	27	26	28	14	0	4	10	
2450^*b*^	Defective	15		96	6		24	7	59												
	Identical			87	0		28	3	56												
	Unique			60	22		8	24	6												
	Overall	57		148	22		37	27	62												
2472	Defective		0	11	0		4	5	2			2	0	1	0	1					
	Identical			29	2		4	8	15			17	0	15	0	2					
	Unique			39	4		20	7	8			11	0	5	3	3					
	Overall		0	75	8		25	18	24			36	1	21	7	7					
2518	Defective			5	0	0	2	0	2	0	1						0				0
	Identical			46	0	0	4	11	19	3	9						0				0
	Unique			72	0	0	21	16	14	2	18						1				1
	Overall			131	0	1	28	28	36	5	33						1				1
2046	Defective			9	0	0	2	2	0	0	5						0				0
	Identical			37	3	0	0	5	22	7	0						0				0
	Unique			58	2	0	13	16	9	4	14						0				0
	Overall			122	5	0	28	25	25	13	26						0				0
2013	Defective			90	0	0	6	3	62	19	0						0				0
	Identical			80	0	0	5	11	50	11	3						0				0
	Unique			39	0	0	5	13	10	10	1						0				0
	Overall			123	0	0	10	26	62	21	4						0				0
2026	Defective			14	4	0	2	3	1	3	1						0				0
	Identical			29	3	2	0	5	22	7	0						0				0
	Unique			63	20	4	10	8	3	17	1						1				1
	Overall			107	23	6	13	13	25	25	2						1				1
Total^*c*^	Defective	60	4	376	39	0	78	53	177	22	7	115	25	38	32	20	11	1	3	7	0
	Identical			528	35	2	55	104	292	28	12	126	16	30	31	49	26	0	8	20	0
	Unique			881	203	4	257	194	176	33	34	475	121	155	121	78	61	3	32	24	2
	Overall	620	87	1,557	259	7	358	335	469	64	65	676	158	201	163	154	96	3	44	47	2

a Pt, participant; PT, pretherapy plasma; OT, on-therapy plasma; R6^+^, X5^−^ R6^+^ T cell subset; X5^+^, X5^+^ R6^−^ T cell subset.

b Source of HIV-1 RNA sequences (17, 23).

c Total number of HIV-1 sequences for all 14 participants from the CHI group.

**FIG 7:**
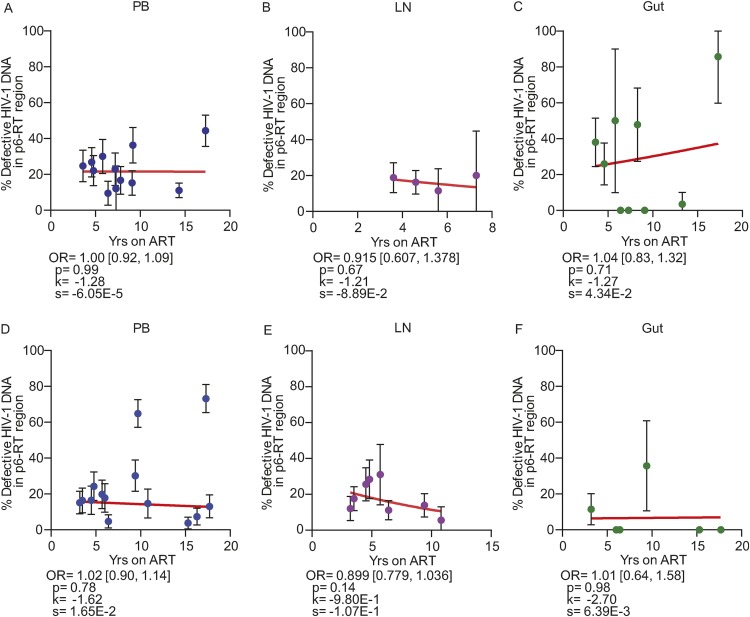
Proportions of defective HIV-1 DNA sequences in the p6-RT region during ART. Shown are the changes in the percentages of defective HIV-1 DNA p6-RT sequences during years on ART in PB (blue circles), LN (pink circles), and gut (green circles) tissues obtained from the AHI group (A to C) and the CHI group (D to F). The changes in the odds that a viral sequence is genetically defective during ART are indicated as OR per year on therapy. The 95% confidence intervals for the ORs are indicated in square brackets. Estimated by mixed-effects logistic regression, the fitted curves (red lines) follow the following equation: *y* = 100 × {1 + exp[−(*k* + *xs*)]}^−1^, where *y* represents the proportions of defective HIV-1 DNA p6-RT sequences, *k* represents the *y* intercept, *s* represents the coefficient calculated as log(OR), and *x* represents the years on ART. Each HIV-1 DNA sequence is the unit of analysis, and the denominator for each sequence is a single HIV-1-infected T cell (33, 73). The confidence intervals of each data point were derived from the binomial distribution. The odds ratios and their confidence intervals were estimated by logistic regression models.

**FIG 8:**
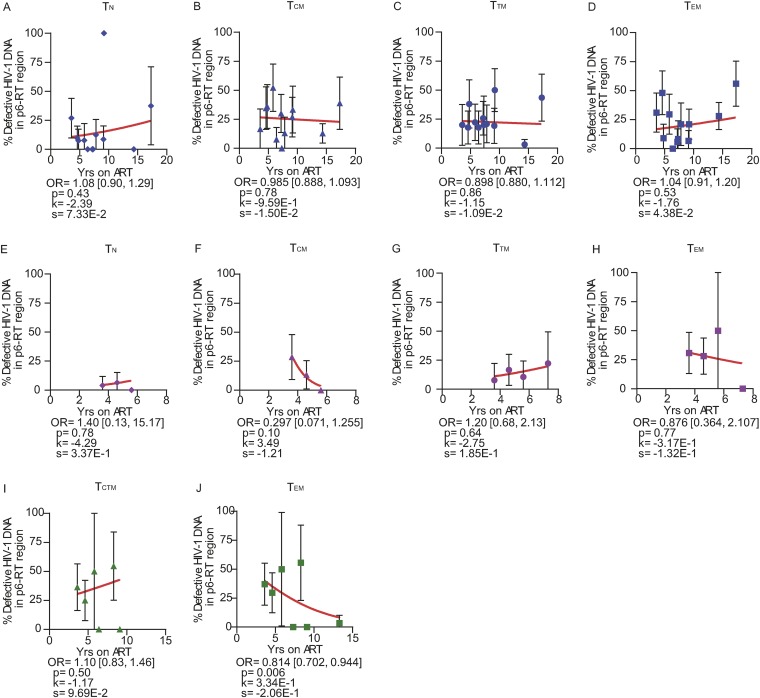
Proportions of defective HIV-1 DNA sequences from CD4^+^ T cell subsets during ART (AHI group). The effects of ART duration on the proportions of defective HIV-1 DNA p6-RT sequences in CD4^+^ T cell subsets sorted from PB (A to D), LNs (E to H), and gut (I to J) are indicated as OR per year on therapy. The 95% confidence intervals for the ORs are indicated in square brackets. Estimated by mixed-effects logistic regression, the fitted curves (red lines) follow the following equation: *y* = 100 × {1 + exp[−(*k* + *xs*)]}^−1^, where *y* represents the proportions of defective HIV-1 DNA p6-RT sequences, *k* represents the *y* intercept, *s* represents the coefficient calculated as log(OR), and *x* represents the years on ART. Each HIV-1 DNA sequence is the unit of analysis, and the denominator for each sequence is a single HIV-1-infected T cell (33, 73). The confidence interval of each data point was derived from the binomial distribution. The odds ratios and their confidence intervals were derived by logistic regression models. Solid blue diamonds, T_N_ cells; solid blue triangles, T_CM_ cells; solid blue circles, T_TM_ cells; solid blue squares, T_EM_ cells, all derived from PB. Solid pink diamonds, T_N_ cells; solid pink triangles, T_CM_ cells; solid pink circles, T_TM_ cells; solid pink squares, T_EM_ cells, all derived from LNs. Solid green triangles, T_CTM_ cells; solid green squares, T_EM_ cells, all derived from the gut.

**FIG 9:**
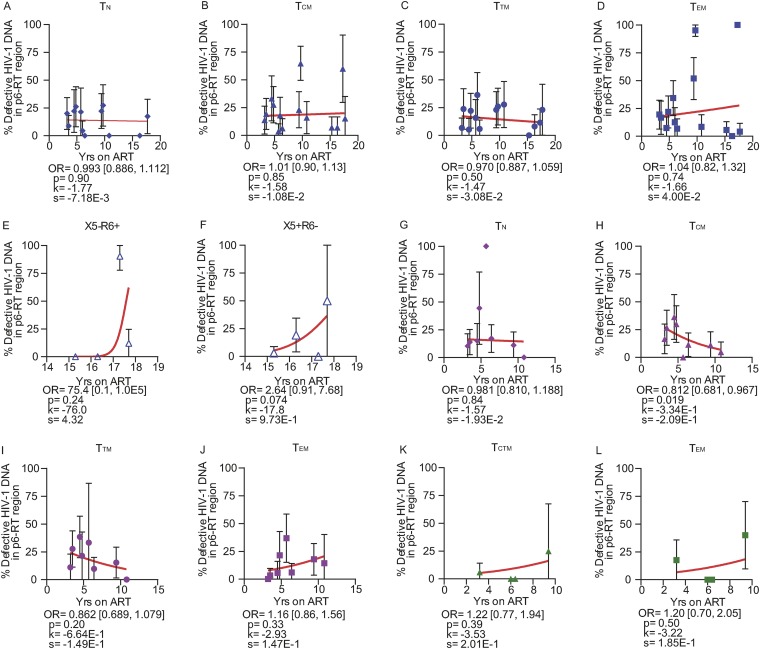
Proportions of defective HIV-1 DNA sequences from CD4^+^ T cell subsets during ART (CHI group). The effects of ART duration on the proportions of defective HIV-1 DNA p6-RT sequences in CD4^+^ T cell subsets sorted from PB (A to F), LNs (G to J), and gut (K to L) are indicated as OR per year on therapy. The 95% confidence intervals for the ORs are indicated in square brackets. Estimated by mixed-effects logistic regression, the fitted curves (red lines) follow the following equation: *y* = 100 × {1 + exp[−(*k* + *xs*)]}^−1^, where *y* represents the proportions of defective HIV-1 DNA p6-RT sequences, *k* represents the *y* intercept, *s* represents the coefficient calculated as log(OR), and *x* represents the years on ART. Each HIV-1 DNA sequence is the unit of analysis, and the denominator for each sequence is a single HIV-1-infected T cell (33, 73). The confidence interval of each data point was derived from the binomial distribution. The odds ratios and their confidence intervals were estimated by logistic regression models. Solid blue diamonds, T_N_ cells; solid blue triangles, T_CM_ cells; solid blue circles, T_TM_ cells; solid blue squares, T_EM_ cells; open blue triangles, gut-homing (X5^−^ R6^+^) cells; open blue triangles, lymph-homing (X5^+^ R6^−^) cells, all derived from PB. Solid pink diamonds, T_N_ cells; solid pink triangles, T_CM_ cells; solid pink circles, T_TM_ cells; solid pink squares, T_EM_ cells, all derived from LNs. Solid green triangles, T_CTM_ cells; solid green squares, T_EM_ cells, all derived from the gut.

Overall, the odds of an HIV-1 DNA sequence being genetically defective did not substantially increase with the duration of ART within PB, LN, and gut tissues obtained from both participant groups (Fig. 7). Similar results were found in specific CD4^+^ T cell subsets sorted from the anatomic sites obtained from the AHI group (Fig. 8) and the CHI group (Fig. 9). Our findings provide evidence that defective HIV-1 DNA p6-RT sequences do not increase substantially in the cell subsets and anatomic sites we analyzed during therapy regardless of when ART was initiated.

### Pre- and on-therapy plasma samples contained few defective HIV-1 RNA p6-RT sequences.

Studies have found that the majority of HIV-1 isolated from virions in the plasma is infectious and replication competent (46 – 48). Therefore, we compared the quantities of defective HIV-1 DNA sequences in T cell subsets sorted from PB, LNs, and gut sampled during ART to the 1,134 HIV-1 RNA sequences isolated from pre- and on-therapy plasma samples in the p6-RT region (Tables 5 and 6).

**FIG 10:**
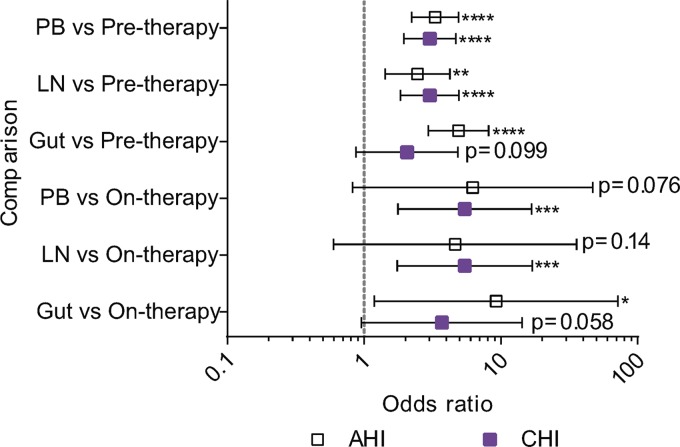
Odds that an HIV-1 sequence was defective in anatomic sites versus plasma samples. Shown is a comparison of the odds that an HIV-1 RNA sequence from pre- and on-ART plasma samples was defective versus the odds that a viral DNA sequence from PB, LN, and gut tissues was defective for the AHI group (open squares) and the CHI group (solid squares). The comparison of the odds is indicated as the OR; the error bars indicate the 95% confidence intervals for the ORs. *, *P* < 0.05; **, *P* < 0.01; ***, *P* < 0.001; ****, *P* < 0.0001. p6-RT sequences were used for the genetic comparisons. The odds ratios and their confidence intervals were estimated by logistic regression models.

HIV-1 DNA sequences derived from the anatomic sites were more often genetically defective than HIV-1 RNA sequences derived from pretherapy plasma samples (Fig. 10). The odds that a PB-derived HIV-1 DNA sequence was genetically defective were about 3-fold higher than those for pretherapy plasma HIV-1 RNA sequences in both participant groups. In LNs, the odds that an HIV-1 DNA sequence was genetically defective were 2.5 and 3.0 times higher than those for pretherapy plasma sequences in the AHI and CHI groups, respectively. In the gut, the odds that a viral DNA sequence was defective were 4.9- and 2.1-fold higher than those for pretherapy plasma RNA sequences in the AHI and CHI groups, respectively. However, they did not reach statistical significance in the CHI group.

**FIG 11:**
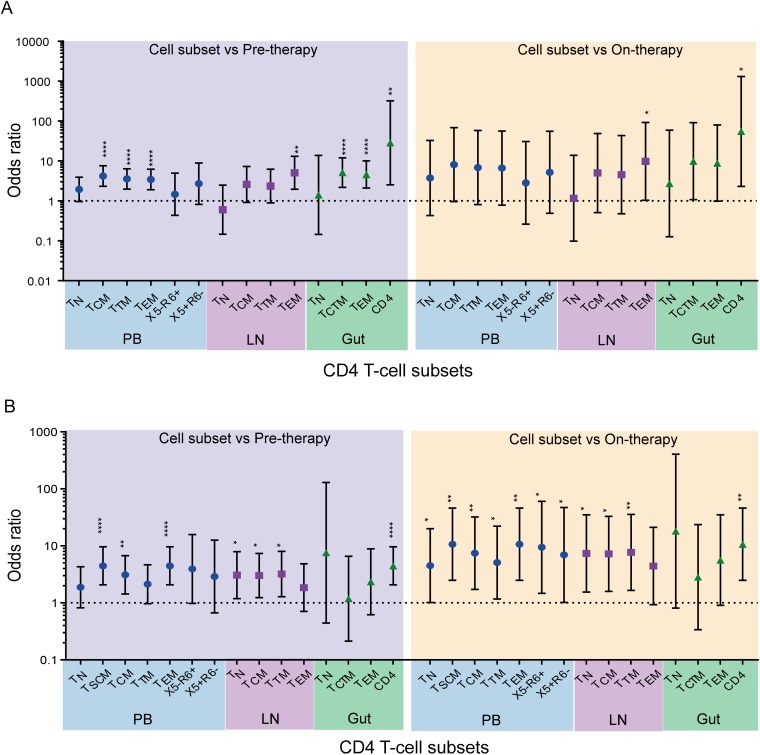
Odds that an HIV-1 sequence was defective in CD4^+^ T cell subsets versus plasma samples. Shown are comparisons of the odds that a viral sequence was defective in CD4^+^ T cell subsets sorted from PB, LN, and gut tissues derived from the AHI group (A) and the CHI group (B) to the odds that they were defective in pre- and on-therapy plasma samples. Blue shading, odds ratio of the CD4^+^ T cell subset to pretherapy plasma; yellow shading, odds ratio of CD4^+^ T cell subset to on-therapy plasma. *, *P* < 0.05; **, *P* < 0.01; ****, *P* < 0.0001. p6-RT sequences were used for the comparisons. The odds ratios and their confidence intervals were derived by logistic regression models.

HIV-1 DNA sequences derived from the anatomic sites contained more genetic defects than viral RNA sequences derived from on-therapy plasma samples, but in some cases, they did not reach statistical significance (Fig. 10). In PB, the odds that a viral DNA sequence was defective were 5- to 6-fold greater than those for on-therapy plasma RNA sequences for both participant groups. Similarly, the odds that a viral DNA sequence was defective were about 5 times higher for LNs than for on-therapy plasma sequences in both participant groups. In the gut, the odds that a viral DNA sequence was genetically defective were 9.2-fold and 3.7-fold higher than for on-therapy plasma HIV-1 RNA sequences derived from the AHI and CHI groups, respectively. Furthermore, a majority of CD4^+^ T cell subsets derived from the anatomic sites also showed higher odds that a viral sequence was genetically defective than pre- and on-therapy plasma-derived sequences in both the AHI and CHI groups (Fig. 11).

### HIV-1 sequences from LN-derived T_EM_ cells were more often genetically identical to pre- and on-therapy plasma viral RNA sequences.

**FIG 12:**
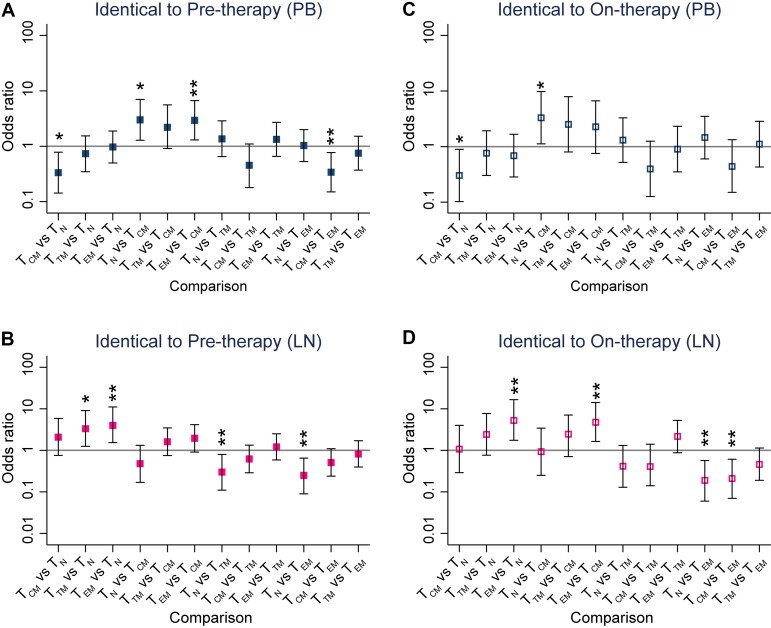
HIV-1 genetic comparisons between pre- and on-therapy plasma samples and T cell subsets from each anatomic site. Shown are comparisons of the odds that an HIV-1 DNA sequence was genetically identical to HIV-1 RNA sequences derived from pretherapy (A and B) or on-therapy (C and D) plasma samples. The odds ratios were derived from every pairwise comparison between CD4^+^ T cell subsets sorted from PB (A and C) and LNs (B and D) obtained from the CHI group. The comparison of the odds is indicated as the OR, and the error bars indicate 95% confidence intervals of the ORs. *, *P* < 0.05; **, *P* < 0.01. p6-RT sequences were used for the genetic comparisons. The odds ratios and their confidence intervals were estimated by logistic regression models.

**FIG 13:**
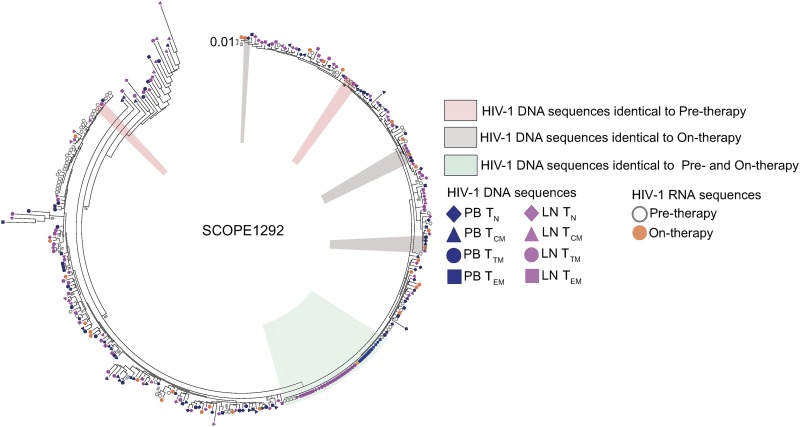
Representative phylogenetic tree showing HIV-1 sequence comparisons between plasma and CD4^+^ T cell subsets. The phylogenetic regions containing HIV-1 DNA p6-RT sequences that were genetically identical to the viral sequences from the pretherapy plasma, to the on-therapy plasma, and to both of the plasma samples are marked. Individual HIV-1 DNA sequences derived from T_N_ (diamonds), T_CM_ (triangles), T_TM_ (circles), and T_EM_ (squares) cells sorted from PB (blue) and LNs (pink) are shown. Individual HIV-1 RNA sequences derived from pre- and on-therapy plasma are also shown. The maximum-likelihood phylogenetic tree was constructed with MEGA6.

We found that HIV-1 RNA p6-RT sequences isolated from pre- and on-therapy plasma contained few genetic defects. The lack of defects within the p6-RT region of HIV-1 RNA sequences derived from pre- and on-therapy plasma indicated that these sequences came from replication-competent virions that could contribute to recrudescence. Therefore, we compared HIV-1 RNA and DNA p6-RT sequences to identify cell subsets that contained viral genomes most closely related to plasma-derived HIV-1. We compared the HIV-1 RNA and DNA sequences from the CHI group (Fig. 12), since HIV-1 sequences in participants treated during chronic infection are genetically diverse (17, 23, 24). A representative phylogenetic tree showing the genetic comparisons between HIV-1 RNA sequences derived from plasma and HIV-1 DNA sequences obtained during ART is presented in Fig. 13. We did not have pre- and on-therapy plasma samples collected from five of the CHI participants. Therefore, nine participants were included in the genetic comparison between HIV-1 DNA sequences isolated from the PB-derived T cell subsets and HIV-1 RNA sequences obtained from pretherapy or on-therapy plasma samples. For the comparison between the LN-derived T cell subsets and the plasma samples, LN tissue was collected from seven participants in the CHI group.

In PB, T_CM_ cells had the lowest odds that an HIV-1 DNA sequence would be genetically identical to a viral RNA sequence derived from pre- and on-therapy plasma samples compared to the other T cell subsets (Fig. 12A and C). In the LNs, T_EM_ cells had 4- and 5-times greater odds that an HIV-1 DNA sequence was genetically identical to viral sequences derived from pre- and on-therapy plasma samples, respectively, than T_N_ cells (Fig. 12B and D). We found that T_EM_ cells had higher odds that a viral sequence was genetically identical to pretherapy and on-therapy plasma HIV-1 RNA sequences than T_CM_ cells. Furthermore, we found 2.2-fold higher odds that a T_EM_-derived HIV-1 DNA sequence was genetically identical to viral sequences derived from on-therapy plasma than T_TM_ cells. Taken together, our genetic comparisons showed that T_CM_ cells within PB were least likely to contain HIV-1 DNA sequences that were genetically identical to HIV-1 RNA sequences derived from pre- and on-therapy plasma samples compared to the other cell subsets. In contrast, LN-derived T_EM_ cells were most likely to contain HIV-1 DNA sequences that were genetically identical to HIV-1 RNA sequences derived from the plasma samples compared to other cell subsets.

### Impact of ART duration on genetically identical and unique HIV-1 DNA sequences.

**FIG 14:**
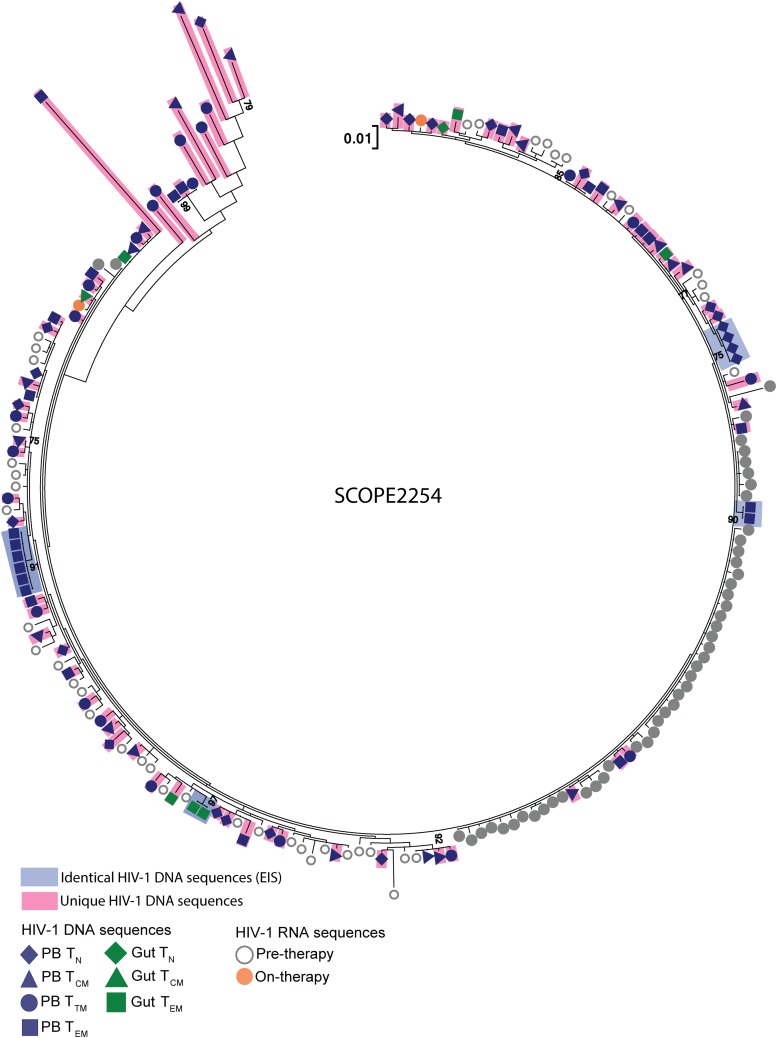
Representative phylogenetic tree showing identical and unique HIV-1 DNA sequences. Individual HIV-1 DNA p6-RT sequences that were genetically unique and located on single branches within a phylogenetic tree are colored pink. The unique viral sequences were genetically distinct and not identical to any other viral RNA and/or DNA sequence. Identical HIV-1 DNA sequences from EIS are colored blue. Individual HIV-1 DNA sequences derived from T_N_ (diamonds), T_CM_ (triangles), T_TM_ (circles), and T_EM_ (squares) cells sorted from PB (blue) are shown. Individual viral DNA sequences derived from T_N_, T_CTM_, and T_EM_ cells sorted from gut (green) are also shown. Individual HIV-1 RNA sequences derived from pre- and on-therapy plasma are shown. The maximum-likelihood phylogenetic tree was constructed with MEGA6.

To assess the contribution of cellular proliferation to viral persistence during ART, we used a mixed-effects logistic regression model to calculate the proportions of genetically identical and unique HIV-1 DNA p6-RT sequences with each additional year of ART for the CHI cohort. As these participants treated during chronic infection contained genetically diverse HIV-1 (17, 23, 24), isolating genetically identical intracellular HIV-1 DNA sequences from these participants suggests the clonal expansion of HIV-1-infected cells. In this study, we define expansions of identical sequences (EIS) as ≥2 genetically identical sequences that are derived from the same cell subset sorted from an anatomic site to identify and quantify cellular proliferation within a specific T cell subset located in a particular anatomic site. Also, we defined unique HIV-1 DNA p6-RT sequences as those that are genetically distinct and exist on a single branch within a phylogenetic tree (Fig. 14). We included both genetically intact and defective HIV-1 DNA sequences in the p6-RT region in the analyses. We analyzed the impact of ART duration on the levels of identical HIV-1 DNA p6-RT sequences, which were derived from EIS, and unique viral sequences to further elucidate the dynamic interplay of HIV-1-infected cells in the viral reservoirs during therapy using mixed-effects logistic regression.

**FIG 15:**
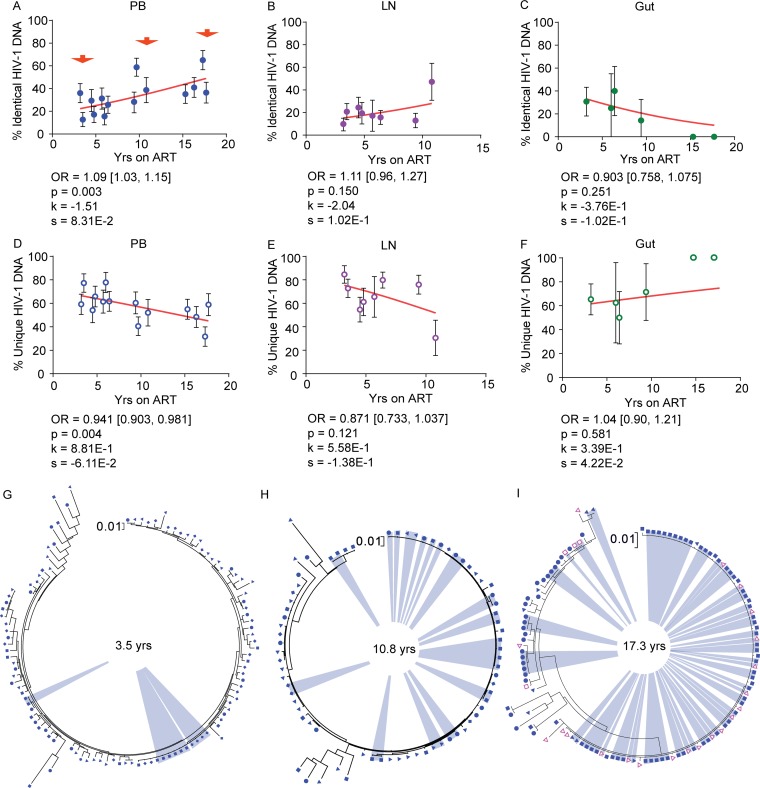
Genetically identical and unique HIV-1 DNA sequences derived from anatomic sites during ART. (A to F) The associations of the percentages of identical (A to C) and unique (D to F) HIV-1 DNA p6-RT sequences with years on ART in PB (A and D), LN (B and E), and gut (C and F) tissues derived from the CHI group are shown. The effects of therapy duration on the odds that a viral sequence was genetically identical are indicated as OR per year on therapy. The 95% confidence intervals for ORs are indicated in square brackets. Estimated by mixed-effects logistics regression, the fitted curves (red lines) follow the following equation: *y* = 100 × {1 + exp[−(*k* + *xs*)]}^−1^, where *y* represents the proportions of genetically identical HIV-1 DNA p6-RT sequences, *k* represents the *y* intercept, *s* represents the coefficient calculated as log(OR), and *x* represents the years on ART. Each HIV-1 DNA sequence is the unit of analysis, and the denominator for each sequence is a single HIV-1-infected T cell (33, 73). The confidence interval of each data point was estimated from the binomial distribution. The confidence interval of each odds ratio was estimated by logistic regression models. (G to I) Maximum-likelihood phylogenetic trees indicating an increase in expansions of genetically identical HIV-1 DNA p6-RT sequences (blue) at 3.5 years (participant 1292) (G), 10.8 years (participant 2472) (H), and 17.3 years (participant 2013) (I) after ART initiation, as indicated in panel A (red arrows). The trees included HIV-1 DNA p6-RT sequences derived from T_N_ (diamonds), T_CM_ (solid triangles), T_TM_ (circles), T_EM_ (solid squares), X5^+^ R6^−^ (open squares), and X5^−^ R6^+^ (open triangles) cells sorted from PB.

We observed that the proportions of identical HIV-1 DNA sequences increased while the proportions of unique viral sequences decreased with duration on therapy in PB and LNs (Fig. 15A, B, D, and E). In the gut, however, we found that the proportions of identical HIV-1 DNA sequences decreased while the proportions of unique viral sequences increased with duration on therapy when data from participants who were on ART for 3 to 17.8 years were included (Fig. 15C and F). However, the trends in LNs and gut did not reach statistical significance. Only PB showed a statistically significant increase in the odds that an HIV-1 DNA sequence was genetically identical to another by a factor of 1.09 per year on ART (95% CI = 1.03 to 1.15; *P* = 0.003) and a decrease in the odds that a viral sequence was unique by a factor of 0.941 (95% CI = 0.903 to 0.981; *P* = 0.004) (Fig. 15A and D). The phylogenetic analysis of HIV-1 DNA sequences derived from PB clearly showed increasing expansions of identical HIV-1 DNA sequences as ART duration increased (Fig. 15G to I).

**FIG 16:**
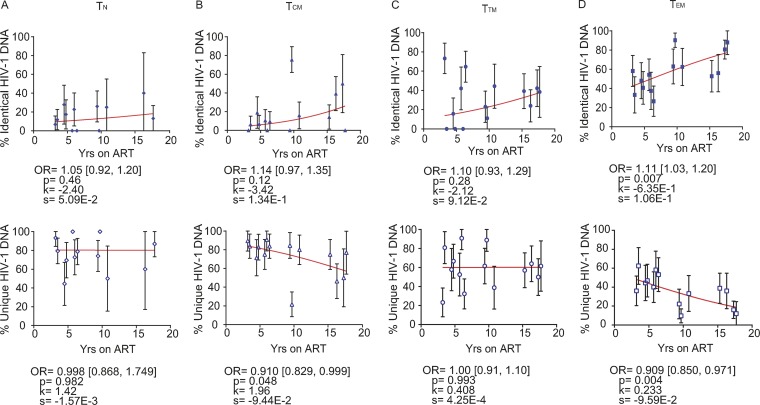
Genetically identical and unique HIV-1 DNA sequences from PB-derived CD4^+^ T cell subsets during ART. The effects of ART duration on the percentages of identical (top) and unique (bottom) HIV-1 DNA p6-RT sequences derived from T_N_ (A), T_CM_ (B), T_TM_ (C), and T_EM_ (D) cells sorted from PB of the CHI group are indicated as OR per year on therapy. The 95% confidence intervals for the ORs are indicated in square brackets. Estimated by mixed-effects logistic regression, the fitted curves (red lines) follow the following equation: *y* = 100 × {1 + exp[−(*k* + *xs*)]}^−1^, where *y* represents the proportions of genetically identical or unique HIV-1 DNA p6-RT sequences, *k* represents the *y* intercept, *s* represents the coefficient calculated as log(OR), and *x* represents the years on ART. Each HIV-1 DNA sequence is the unit of analysis, and the denominator for each sequence is a single HIV-1-infected T cell (33, 73) The confidence interval of each data point was estimated from the binomial distribution. The odds ratios and their confidence intervals were estimated by logistic regression models.

**FIG 17:**
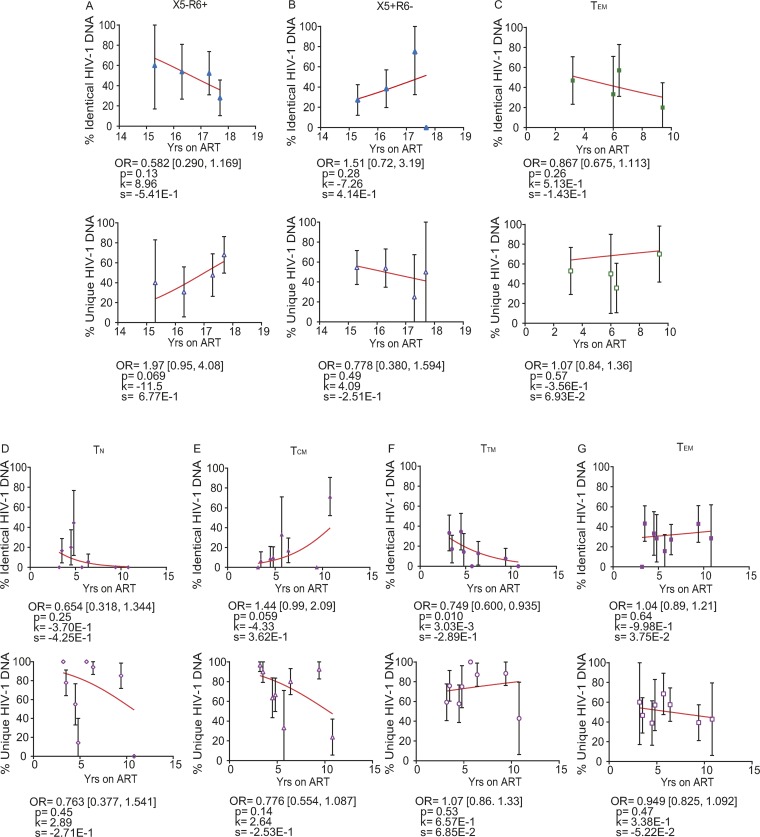
Genetically identical and unique HIV-1 DNA sequences from tissue-homing and tissue-derived CD4^+^ T cell subsets during ART. The effects of ART duration on the percentages of identical (top) and unique (bottom) HIV-1 DNA p6-RT sequences derived from CD4^+^ T cell subsets sorted from PB, LN, and gut tissues obtained from the CHI group are indicated as OR per year on therapy. The effects of ART duration on the proportions are shown in X5^−^ R6^+^ (A) and X5^+^ R6^−^ (B) cells sorted from PB; in T_EM_ cells (C) sorted from the gut; and in T_N_ (D), T_CM_ (E), T_TM_ (F), and T_EM_ (G) cells sorted from LNs. The 95% confidence intervals for the ORs are indicated in square brackets. Estimated by mixed-effects logistic regression, the fitted curves (red lines) follow the following equation: *y* = 100 × {1 + exp[−(*k* + *xs*)]}^−1^, where *y* represents the proportions of genetically identical or unique HIV-1 DNA p6-RT sequences, *k* represents the *y* intercept, *s* represents the coefficient calculated as log(OR), and *x* represents the years on ART. Each HIV-1 DNA sequence is the unit of analysis, and the denominator for each sequence is a single HIV-1-infected T cell (33, 73). The confidence interval of each data point was derived from the binomial distribution. Odds ratios and their confidence intervals were estimated by logistic regression models.

We observed that genetically identical HIV-1 DNA sequences increased whereas unique viral sequences decreased in a majority of CD4^+^ T cell subsets sorted from PB, LNs, and gut during therapy (Fig. 16 and 17). In PB-derived T_EM_ cells, however, we observed the strongest evidence that the proportions of identical HIV-1 DNA sequences increased while the proportions of unique viral sequences decreased during each additional year on therapy (Fig. 16D). The odds of an HIV-1 DNA sequence being genetically identical to another in T_EM_ cells increased with the duration of therapy by a factor of 1.11 per year (95% CI = 1.03 to 1.20; *P* = 0.007) (Fig. 16D). The odds that a viral sequence would be unique, however, decreased per year on ART by 0.909-fold in T_EM_ cells (95% CI = 0.850 to 0.971; *P* = 0.004) (Fig. 16D).

## DISCUSSION

Understanding the impact of ART duration on the dynamics and genetic composition of HIV-1-infected cells is critical for effective curative strategies. We therefore performed detailed genetic analyses of HIV-1 RNA sequences derived from pre- and on-therapy plasma samples and HIV-1 DNA sequences derived from a broad range of CD4^+^ T cell subsets sorted from blood, lymph node, and gut tissues from participants who initiated treatment during acute/early and chronic infection. These studies allowed us to elucidate how the duration of therapy affects the number of HIV-1-infected T cells and the genetic composition of the proviruses they contain. In this cross-sectional analysis, we analyzed samples obtained after at least 3 years of effective therapy when HIV-1 replication was fully suppressed to below the limit of detection to minimize the impact of episomal HIV-1 DNA in the single-proviral sequencing (SPS) data (49, 50).

We investigated the genetic composition of HIV-1 within the p6-RT region. It is well known that no subgenomic region can accurately represent the genetic diversity of full-length HIV-1 sequences (43, 44, 51). However, it has been shown that the *gag*-p6-*pro* region is better than other subgenomic regions at predicting the genetic diversity of full-length HIV-1 genomes in the viral populations derived from participants who initiated therapy during acute and chronic infection (51). The HIV-1 gene region we analyzed was longer than *gag*-p6-*pro* and had a clonal prediction score of 95, meaning that identical sequences in this region are identical throughout the viral genome with a probability of 95% (51). We analyzed over 5,000 HIV-1 RNA and DNA sequences, including both genetically intact and defective sequences, in this p6-RT region. Therefore, the subgenomic region selected for our genetic analyses, the clonal prediction score of the region, and the large number of sequences we analyzed increase the likelihood that the results of our study reflect the findings that would have resulted from full-length HIV sequence analyses. Moreover, the p6-RT HIV-1 DNA sequences that contain defects are not functional, as the region encodes viral enzymes important for replication. Previous studies have shown that defective HIV-1 genomes can produce viral proteins, including gag and pol (52, 53). Also, CD4^+^ T cells from HIV-1-infected participants showed an increase in the expression of p24 when incubated with gag and pol viral proteins, indicating that these viral proteins can induce viral replication within infected cells (54). The p6-RT region we sequenced includes *gag* and *pol*, and based on the studies described above, we believe that quantifying how the proportions of defective or intact p6-RT sequences change over the duration of ART can help assess the risk of viral rebound and spread when ART is interrupted.

Several studies have demonstrated that the number of HIV-1-infected cells decays more rapidly in HIV-1-infected individuals who initiated therapy during early infection than in those who started treatment during chronic infection (55 – 57). Within the peripheral blood derived from the AHI group, we found an 18% reduction in the proportions of cells infected during each additional year of therapy. This result was similar to that of a recent longitudinal study that estimated the decay rate within CD4^+^ T cells from peripheral blood at 13% per year on ART (55). However, one of the studies included participants who had several episodes of intermittent viremia per year, indicating that their infections were not fully and continuously suppressed by the treatment (57). Moreover, the studies assessed the virus from total CD4^+^ T cells (55, 57) or sorted a limited population of memory CD4^+^ T cells (56) from peripheral blood when demonstrating a decay of HIV-1-infected cells during therapy. In contrast to these previous findings, we found similar decreases in the number of HIV-1-infected cells within the peripheral blood from both participant cohorts irrespective of whether treatment was initiated during acute/early or chronic infection. In both participant cohorts, this decrease in HIV-1-infected cells resulted from a statistically significant association of lower HIV-1 infection frequencies of naive and central memory T cells with each additional year of ART. However, in the case of participants treated during chronic infection, we found a large decrease in the proportion of HIV-1-infected cells over the years of therapy in the CXCR5^+^ CCR6^−^ cell subset. A recent study describes cells expressing PD-1 and CXCR5 as circulating follicular helper T cells (58). We did not sort for the PD-1 cell marker, but our data may suggest that HIV-1 infection of circulating follicular helper T cells is less stable and even decays over time. Moreover, it has been found that CXCR5^+^ memory CD4^+^ T cells sorted from peripheral blood coexpress CCR7, indicating these cells comprise a subpopulation of peripheral-blood-derived T_CM_ cells (59). Thus, the CXCR5^+^ CCR6^−^ cell subset could also have contributed to the decrease in the number of HIV-1-infected T_CM_ cells during each year on therapy in the peripheral blood of the participants treated during chronic infection. Overall, these findings indicate that the numbers of HIV-1-infected naive and T_CM_ cells within the peripheral blood decrease during effective therapy. This observed decrease could be due to the decay of T cells that harbor defective proviruses, as cells contributing to the HIV-1 reservoir are known to contain predominantly defective viral genomes (44).

For AHI and CHI participants with PB and LN samples available for analysis, HIV-1-infected cells from the lymph node decreased per year on ART. A substantial decrease in the proportion of HIV-1-infected T cells during each additional year on ART was found for all lymph node-derived CD4^+^ T cell subsets from four participants treated during acute/early infection with paired PB and LN samples. Extrapolation to all 12 acute/early participants estimated smaller reductions in the proportion of HIV-1-infected cells within LNs over 3 to 17.8 years of therapy that were similar to those for the participants treated during chronic infection. This finding suggests that tissue restoration and T cell reconstitution in lymph nodes reduced the number of HIV-1 cells during therapy for both participant groups (60).

Due to errors made during HIV-1 replication, genetic mutations that reduce viral fitness accumulate (15, 37, 38, 61). Genetic defects, such as G-to-A hypermutation, internal deletions, and nucleotide insertions and/or deletions that cause a frameshift, also accumulate in proviruses during viral replication (15, 37 – 42, 45, 62). However, our previous studies have shown a lack of HIV-1 evolution during ART, supporting a small amount of evidence for ongoing viral replication during therapy (17, 23). We combined all the defective features within the p6-RT region and found that the proportion of genetically defective HIV-1 DNA sequences did not appear to increase substantially with the duration of therapy. This was observed in most of the cell subsets and the anatomic sites we analyzed regardless of when ART was initiated. Our results revealing that defective HIV-1 sequences do not accumulate during therapy agree with those of a recent study showing that cytotoxic T cells can target cells containing defective proviruses (63). Taken together, our findings provide evidence that defective viral sequences are established during multiple rounds of viral replication before viral suppression rather than during effective therapy.

We found that plasma samples obtained before ART and during the early phase of treatment contained a lower proportion of defective viral sequences than HIV-1 DNA sequences derived from blood or tissue CD4^+^ T cells. This result indicates that plasma-derived viral sequences most likely represent a population of HIV-1 that is infectious and capable of producing new virions (46 – 48). Compared to intracellular HIV-1 sequences from the other T cell subsets, T_CM_ cells from the peripheral blood were least likely to contain HIV-1 DNA sequences that were identical to pre- and on-therapy plasma RNA sequences. Our recent study revealed that T_CM_ cells contain the smallest amount of genetically intact HIV-1 proviruses in peripheral blood (43). In agreement with the previous study, our genetic analysis involving intracellular HIV-1 DNA sequences and plasma-derived viral RNA sequences suggests that the T_CM_ cell subset within the peripheral blood is the least likely cellular source for infectious HIV-1. Importantly, our genetic analyses revealed that, in the lymph node tissue, T_EM_ cells were highly enriched with viral sequences that were genetically identical to plasma-derived HIV-1 sequences. These results suggest that T_EM_ cells are a probable source for infectious HIV-1 in the lymph node compared to all other T cell subsets.

The presence of genetically identical HIV-1 DNA sequences indicates that cellular proliferation plays a role in the maintenance of persistent HIV-1 during therapy (17, 23, 25 – 28, 43, 64). Our in-depth genetic analysis revealed that the genetically identical HIV-1 DNA sequences increased whereas the unique viral sequences decreased in a majority of CD4^+^ T cell subsets sorted from PB and LNs during therapy. This indicates that cellular proliferation compensates for the decay of HIV-1-infected CD4^+^ T cells in the peripheral blood and lymph nodes over time.

We found that expansions of genetically identical HIV-1 DNA sequences increased over the years of therapy at a statistically significant rate in the peripheral blood and that peripheral-blood-derived T_EM_ cells were the main contributors. This is in agreement with our previous longitudinal study, which showed that identical HIV-1 DNA sequences increased in PB-derived T_EM_ cells over 6 months of therapy in both p6-RT and *env* genomic regions (17). In contrast, the population of unique HIV-1 DNA sequences decayed in peripheral blood at a statistically significant rate, particularly in T_EM_ cells. A previous study found that an increase in the proportion of identical HIV-1 integration sites was associated with a decrease in the proportion of unique viral integration sites in PB-derived CD4^+^ T cells during therapy (65). This supports the inverse relationship between identical and unique p6-RT HIV-1 DNA sequences within PB, particularly within the PB-derived T_EM_ cell subset. Also, it has been noted that the change in the frequency of PB-derived T_EM_ cells containing HIV-DNA was less than 2-fold during 6 months of effective therapy (17). In agreement with this previous study, we found that the HIV-1 infection frequency appeared to be stable during 3 to 18 years of therapy in T_EM_ cells sorted from peripheral blood. Taken together, our findings provide strong evidence that the overall stability of HIV-1-infected T_EM_ cells is regulated by cellular proliferation that restores T cell loss during therapy with the clonal expansion of particular T_EM_ cells containing identical HIV-1 sequences (possibly in response to an antigen) and the reduction/extinction of T_EM_ cells containing unique HIV-1 sequences. The one anatomic region that revealed a decrease in identical sequences and an increase in unique sequences was the gut; however, the findings in the gut did not reach statistical significance. This suggests that cells located in the gut are under strict immune regulation preventing their response to normal gut flora, which limits cellular proliferation and expansion of identical HIV-1 sequences (66 – 70).

Although there are limitations to cross-sectional analyses, this study of more than 5,000 HIV-1 single-genome sequences from 26 participants has provided important insights into the HIV-1 reservoir during 3 to 17.8 years of therapy. A longitudinal study using full-length HIV-1 sequencing and integration site analysis of individual participant samples over time would be ideal to reconfirm our findings, but it would be time-consuming and costly (43, 44, 64, 65, 71, 72). Moreover, the number of cells required to adequately sort specific T cell subsets for full-length HIV-1 sequencing and integration site analysis would limit the ability to conduct such a study using frozen peripheral blood cells and tissue samples, which most laboratories have for longitudinal samples.

In conclusion, our in-depth genetic characterization of HIV-1 within anatomic sites after 3 to 17.8 years of ART revealed several important findings. The number of HIV-1-infected memory T cells decays in all anatomic sites analyzed with early and late ART initiation. The absence of substantial increases in the pool of defective HIV-1 sequences during effective therapy in both participant cohorts suggests that the defects are established during multiple rounds of viral replication before viral suppression rather than during effective ART. Moreover, lymph node-derived T_EM_ cells are a probable source of HIV-1 genomes capable of producing infectious virus. Importantly, the complex interplay of identical and unique HIV-1 DNA sequences indicates that cellular proliferation plays a significant role in the maintenance of persistent HIV-1, and these mechanisms are most pronounced in peripheral-blood-derived T_EM_ cells.

## MATERIALS AND METHODS

### Study approval.

This project was approved by the institutional review board at the Western Sydney Health Department for the Westmead Institute for Medical Research (AU RED LNR/13/WMEAD/315) and the ethics review committees at the University of California San Francisco (UCSF) (10-01330/068192 and 10-02631/083640) and the Vaccine Gene Therapy Institute—Florida (VGTI-FL) (FWA 00004139). All participants provided written informed consent prior to inclusion in the study.

### Participant selection.

HIV-1-infected adults on effective ART for 3.0 to 17.8 years were recruited for the study at UCSF, San Francisco, CA, USA. The inclusion criteria for the study were at least 3 continuous years of therapy, with undetectable viral loads since month 6 of therapy and HIV-1 RNA at <40 copies/ml for at least 3 years. All the participants had viral loads of <40 HIV-1 RNA copies/ml during therapy except for participant 2450, whose viral load rebounded to 3,418 HIV-1 RNA copies/ml at the time of sampling (Table 1).

### Clinical samples.

PB samples from 26 participants infected with HIV-1 subtype B who were on long-term suppressive ART (duration, 3.0 to 17.8 years) were analyzed. Of those participants, LN and gut (gut-associated lymphoid, ileum, and/or rectum) tissues were available from 12 and 17 participants, respectively. Samples were collected from 12 participants who initiated therapy ≤6 months after infection (acute/early) and 14 participants who initiated therapy ≥1 year after HIV-1 infection (chronic) (Table 1). Note that all the clinical samples analyzed were collected after the stated duration of therapy for each participant (Table 1). The CD4 counts of all participants ranged from 339 to 1,165 cells/μl at the time of sample collection (Table 1). We completed and published in-depth longitudinal genetic characterizations of the HIV-1 of 8 participants (Tables 5 and 6) (17, 23). Here, we conducted an interpatient cross-sectional study of the impact of treatment duration on the HIV-1 reservoir, which included our published data (17, 23).

### Isolation of cells from peripheral blood, lymph node, and gut tissues.

The T cell subsets were isolated from peripheral blood, lymph node, and gut tissues as previously described (17, 23). Briefly, 230-ml peripheral blood samples were collected in tubes containing acid citrate dextrose as an anticoagulant or by leukapheresis. Peripheral blood mononuclear cells (PBMCs) were separated from plasma using Ficoll within 30 min after collection. Total CD4^+^ T cells were isolated from PBMCs using magnetic negative selection (Stem Cell Technologies, Vancouver, Canada) according to the manufacturer’s protocol. For isolation of cells from lymph nodes, 1/2 to 1 inguinal lymph node was removed under local anesthesia. Mechanical dissociation, followed by filtration and washing, was applied to the lymph node tissue. The isolation of cells from gut tissues and rectal and ileal biopsy specimens was accomplished using enzymatic digestion of Liberase TL or Liberase DL (Roche), respectively, in association with DNase I (Sigma) and mechanical disruption using GentleMacs (Miltenyi).

### Cell sorting.

Fluorescence-activated cell sorting (FACS) (FACSAria; BD Biosciences) was used to sort CD4^+^ T cell subsets, as previously described (17, 23). CD4^+^ T_N_, T_CM_, T_TM_, and T_EM_ cells were sorted from peripheral blood sampled from all 26 participants (Tables 1 to 3 and Fig. 1) (17, 23). CD4^+^ T_SCM_, memory CXCR5^−^ CR6^+^ (X5^−^ R6^+^) and X5^+^ R6^−^ T cell subsets were sorted from blood samples from 6 participants whose blood samples were collected after at least 15 years of therapy (Tables 1 to 3 and Fig. 1 and 2). CD4^+^ T_N_, T_CM_, T_TM_, and T_EM_ cells were sorted from lymph nodes obtained from 12 participants (Tables 1 to 3 and Fig. 3) (17). Figures 1 to 3 show representative cell-sorting strategies from PB and LNs. CD4^+^ T cells were sorted from gut tissues of 5 participants whose gut samples were collected after >15 years of therapy (participants 2115, 2518, 2046, 2013, and 2026) (Tables 1 to 3). CD4^+^ T_N_, T_CTM_, and T_EM_ cell subsets were derived from gut samples obtained from another 12 participants using a gating strategy published previously (Tables 1 to 3) (17, 23). For peripheral blood, lymph node, and gut samples, the monoclonal antibodies used for cell sorting were CD3-V450, CD4-phycoerythrin (PE) Cy7, CD45RO-ECD, CD27-allophycocyanin (APC) AeF780, CCR7-Alexa 647, CD57-fluorescein isothiocyanate (FITC), CD45-Alexa 700, CD13-PE, and CD14-Qdot 605. Dead and live cells were distinguished with aqua-fluorescent reactive dye (ARRD). The monoclonal antibodies were obtained from Invitrogen and BD Biosciences/Pharmigen (17). For leukapheresis samples, the monoclonal antibodies used for cell sorting were CD3-Alexa 700 (clone UCHT1; BD no. 557943), CD4-APC (clone RPA-T4; BD no. 555349), CD14-V500 (clone M5E2; BD no. 561391), LIVE/DEAD aqua marker (Invitrogen no. L34957), CD45RA-BV650 (clone HI100; BioLegend no. 304135), CD45RO-peridinin chlorophyll protein (PerCP) eFluor710 (clone UCHL1; eBioscience no. 40-0457-42), CD27-APC eFluor780 (clone O323; eBioscience no. 47-0279-42), CCR7-PE CF564 (clone 15053; BD no. 562381), CD127-PE (cloneHIL-7R-M21; BD no. 557938), CD95^−^ PECy7 (clone DX2; BD no. 561633), CCR6^−^ PE (clone 11A9; BD no. 559562), and CXCR5-Alexa 488 (clone RF8B2; BD no. 558112). The detailed gating strategy is presented in Fig. 1 to 3. From rectal and ileal tissues, CD4^+^ T cells were sorted from single-cell suspensions using the following combinations of antibodies: CD3-Alexa 700 (clone UCHT1; BD no. 557943), CD4-APC (clone RPA-T4; BD no. 555349), CD8-PB (clone RPA-T8; BD no. 557943), CD14-V500 (clone M5E2; BD no. 561391), and LIVE/DEAD aqua marker (Invitrogen no. L34957). The markers used for sorting each CD4^+^ T cell subset from each anatomic site are presented in Table 3. For each cell type derived from peripheral blood, lymph nodes, and gut, 62 × 10^6^ to 30 × 10^6^ cells were sorted into FACS tubes. The cells were divided and spun down in 1.5-ml Eppendorf tubes. The supernatant was removed, and the cell pellets were stored at −80°C for further analysis. Postsort analysis of cellular purity was done for each cell type from peripheral blood, with means of 93.4% for T_N_, 90.3% for T_CM_, 90.9% for T_TM_, 95.3% for T_EM_, 97.3% for CXCR5^−^ CCR6^+^, and 94.9% for CXCR5^+^ CCR6^−^ cell subsets. The cellular purity for T_N_ and CD4^+^ memory T cell subsets sorted from lymph node tissue and gut was similar to the previous report (17). The purity of CD4^+^ T cells sorted from gut tissues was 94.9%. The cell purity for T_SCM_ cells was not assessed due to the low number of cells sorted. For HIV-1 sequencing, we analyzed an average of 5.0 × 10^3^ to 2.4 × 10^6^ cells in each CD4^+^ T cell subset (Table 2).

### DNA extraction.

DNA was extracted from CD4^+^ T cell subsets sorted from peripheral blood and gut samples collected from 6 participants who were on ART for at least 15 years (Table 1). Four hundred microliters of RNAzol RT (MRC, Inc.) was added to a 1.5-ml Eppendorf tube containing the cell pellet; a 0.4× RNAzol RT volume of sterile nuclease-free water (Invitrogen) was added and mixed by inversion for 15 s, followed by incubation for 15 min. The mixture was centrifuged at 16,000 × *g* for 15 min at room temperature. The top phase was removed, and the bottom phase was used for DNA extraction. Nine hundred microliters of DNAzol (MRC, Inc.), followed by 10 μl of glycogen (20 μg/μl; Qiagen), was added to the bottom phase. DNA was precipitated by adding 500 μl of 200 proof ethanol (Sigma-Aldrich). The mixture was incubated for 10 min at room temperature and centrifuged at full speed for 30 min. The supernatant was removed, and the DNA pellet was washed with 75% ethanol twice. The pellet was air dried until no ethanol was visible. The pellet was dissolved in 300 μl of 8 mM NaOH (Sigma-Aldrich), followed by neutralization by adding 24 μl of 0.1 M HEPES (Gibco).

### Single-proviral sequencing.

The SPS assay was developed to obtain many individual intracellular HIV-1 DNA sequences from cells of participants on long-term therapy to assess the viral DNA population characteristics, diversity, evolution, and HIV infection frequencies of the cells (17, 23, 33, 73). We validated the technique using tissue culture cells with known numbers of genetically distinct HIV-1 proviruses. We applied this proven technique to quantify and genetically characterize HIV-1 DNA sequences from T cell subsets. Using advanced fluorescence-activated cell sorting, CD4^+^ T_N_, T_SCM_, T_CM_, T_TM_, T_EM_, and memory X5^+^ R6^−^ and X5^−^ R6^+^ cells were sorted from peripheral blood and lymph node tissue samples based on their cellular phenotypes (Fig. 1 and 3 and Table 3) (17, 23, 74). From gut tissues, we sorted CD4^+^ T_N_, T_CTM_, T_EM_, and total CD4^+^ T cells (Table 3). Briefly, the lysate of the sorted cells or the extracted DNA was serially diluted (1:1 to 1:729). Single HIV-1 DNA molecules were amplified using primers flanking the *gag-pol* region (p6-RT). PCR amplification and sequencing of the DNA molecules in each well allowed quantification and analysis of the genetic relationship of HIV-1 DNA sequences in each infected cell subset.

### Single-genome sequencing.

Plasma was collected before and during ART, and HIV-1 RNA sequences were obtained using single-genome sequencing (SGS). The HIV-1 RNA sequences were compared to the HIV-1 DNA sequences identified using SPS, as described previously (75 – 77). At least 22 ml of plasma was used to pellet the virus. The pre-ART plasma samples were collected from 20 out of 26 participants from 4.7 years before to just prior to the initiation of treatment (Table 1) (17, 23). Eighteen out of 26 participants had plasma samples collected after ART initiation (Table 1). The on-therapy plasma samples from 5 previously reported participants were collected at 5.8 to 13.4 years of ART (17, 23). The on-therapy plasma samples from the remaining 12 participants were collected at ≤3 months after the initiation of therapy (Table 1). Only the sequences from the CHI group were used for the comparison of HIV-1 RNA sequences derived from pre- and on-ART plasma samples and HIV-1 DNA sequences obtained from CD4^+^ T cell subsets sorted from different anatomic sites during therapy.

### Phylogenetic analyses.

Using previously described methods (17, 23), HIV-1 sequences derived from plasma and CD4^+^ T cell subsets were phylogenetically analyzed. The SPS and SGS methods preclude the resampling of HIV-1 DNA and RNA sequences, respectively. The sequence data were generated over 3 to 4 years in two different physical locations with protocols designed to prevent cross-contamination between clinical samples and laboratory-related HIV strains. p6-RT sequences were aligned using MAFFT (78). Hypermutants (HIV-1 sequences containing G-to-A hypermutations) were identified using the Los Alamos Hypermut tool (79). Sequences containing premature stop codons, insertions/deletions causing a frameshift, and/or internal deletions were identified by manual screening and the Los Alamos quality control tool. The HIV-1 RNA sequences derived from pre-ART and early on-ART plasma samples and HIV-1 DNA sequences derived from a broad spectrum of CD4^+^ T cell subsets sorted from PB, LN, and gut tissues were used to construct maximum-likelihood phylogenetic trees for each participant using MEGA 6 (1,000 bootstrap replicates; general time-reversible substitution model with gamma distribution and proportion of invariant sites; gamma category 4) (80). The phylogenetic trees containing all viral sequences were used to visually locate EIS (80). The genetically identical HIV-1 DNA sequences included at least 2 viral DNA sequences with 100% pairwise identity and were found in monotypic groups of a phylogenetic tree without any internal branches. Also, these identical HIV-1 DNA sequences were derived from a specific CD4^+^ memory T cell subset sorted from an anatomic site. Maximum-likelihood phylogenetic trees at 3 different time points on ART (3.5, 10.8, and 17.3 years, respectively) were constructed to visualize the number of EIS from PB-derived intracellular HIV-1 DNA sequences at these specific time points. The unique HIV-1 DNA sequences are located on single branches within a phylogenetic tree and are genetically distinct and not genetically identical to any other viral RNA and/or DNA sequence (Fig. 14). The denominator for identical and unique HIV-1 DNA sequences, whether they are defective or not, is a single infected T cell. This is due to the fact that more than 90% of the HIV-1-infected T cells contain a single HIV-1 DNA molecule when analyzed by single-cell sequencing (33, 73). Previously published HIV-1 DNA and RNA sequence are available in GenBank (accession no. KP065816 to KP067089, KP113063 to KP113482, KP152533 to KP152580, and KP152658 to KP153066) (17).

### Statistics.

We used previously described maximum-likelihood methods (23) to estimate the proportion of infected cells in each cell population in each tissue type from each participant. The maximum-likelihood method was used to calculate the proportion of infected cells that would be most consistent with the observed numbers of PCR-positive wells at different dilutions with various total numbers of cells (81). We then created simpler “stand-in” data to convey equivalent information for further analysis. For each cell population from a given tissue type in a given participant, we defined stand-in data as *r* infected cells out of *n* examined, with *r* and *n* chosen so that a Poisson regression model with only an intercept term would reproduce the same estimated infection rate and confidence interval around it as obtained in the maximum-likelihood analyses. When no wells were positive, we set *r *equal to 0 and *n* equal to the total number of cells tested. We performed statistical analyses of these data using negative binomial regression, because this generalizes Poisson regression to allow additional variation. We used the SAS NL mixed procedure (SAS Institute, Cary, NC; version 9.4) to fit negative binomial regression models for each cell population from each tissue with a linear effect of years on ART, which estimates the fold effect per year on therapy. The fold effect per year on therapy is a multiplicative effect that is equivalent to the fold change in the proportion of HIV-1-infected T cells from earlier to later time points. We summarized the results across cell populations within each tissue type with geometric means. The intercept and slope coefficients from the models were used to fit curves to the number of HIV-1-infected T cells per million over years on ART, as shown in equation 1:

(1) $$y=1,000,000\times e^{\left(k+\mathrm{xs}\right)}$$

where *y* is the number of HIV-1-infected T cells per million, *k* is the intercept, *s* is the slope per year on ART, and *x* is the number of years on ART. This statistical calculation was applied when at least four participants contributed to the fold effect per year on ART within each T cell population and tissue.

To obtain a *P* value for the differences in rates of decline, we fitted a mixed-effects negative binomial regression model to the PB and LN data together, with a random intercept to account for within-person correlation of HIV-1 infection rates in PB and LN samples of the same population from the same person. These unexpectedly showed nonsignificant *P* values and very different estimates of the fold effect per year of ART in LNs in the AHI group, suggesting that the 4 participants with LN samples available might not be representative of the entire set of 12. The mixed-effects models used the within-person PB-LN correlations to estimate fold effects for LNs that are less susceptible to bias caused by having the data from only four participants of the AHI group (the extrapolated estimates in Fig. 4). We calculated the Pearson correlations of the logarithmically transformed proportions of T cells that were HIV-1 infected, specifically using the following formula: log[max(*r*,0.5)/*n*]. When estimated proportions were zero, we changed *r* to 0.5 in order to permit logarithmic calculation. We excluded gut measurements based on fewer than 1,000 cells when performing the Pearson correlations, because they were likely to be imprecise.

We defined dichotomous dependent variables for each genetic sequence of HIV-1 RNA derived from pre- and on-ART plasma samples and HIV-1 DNA sequences derived from CD4^+^ T cell subsets sorted from peripheral blood, lymph node, and gut tissues obtained after 3 to 17.8 years of ART. These variables indicated whether a viral sequence was genetically defective, unique, or coming from EIS. An additional variable indicated whether an HIV-1 DNA sequence had an identity of 100% compared to a viral sequence derived from pre- or on-ART plasma samples (82). We applied mixed-effects logistic models with random-person effects to compare the odds of a viral sequence being genetically defective between the plasma samples, the anatomic sites, and the CD4^+^ T cell subsets. We applied similar models to compare the odds of an HIV-1 DNA sequence being genetically identical to an HIV-1 RNA sequence derived from pre- or on-ART plasma samples, using CD4^+^ T cell subsets (T_N_, T_CM_, T_TM_, and T_EM_) of peripheral blood and lymph nodes as the categorical predictor variable. For analyses involving the duration of ART, we applied mixed-effects logistic regression to estimate the odds ratio (OR) per year on ART. We plotted the proportions of defective, unique, or identical HIV-1 DNA sequences across ART duration and fitted nonlinear curves derived from mixed-effects logistic regression models using equation 2 below;

(2) $$y=100\times \frac{1}{1+e^{-\left(k+\mathrm{xs}\right)}}$$

where *y* is the fitted percentage of defective, unique, or identical HIV-1 DNA sequences; *k* is the intercept; *s* is the log odds ratio per year on ART; and *x* is the number of years on ART. The fitted curves were derived from the models, with a viral sequence as the unit of analysis. Moreover, the curves account for the plotted confidence intervals, which include the number of sequences available for each time point.

### Accession number(s).

The HIV-1 DNA and RNA sequences have been submitted to GenBank under accession numbers MH830518 to MH834389.
